# ALS monocyte-derived microglia-like cells reveal cytoplasmic TDP-43 accumulation, DNA damage, and cell-specific impairment of phagocytosis associated with disease progression

**DOI:** 10.1186/s12974-022-02421-1

**Published:** 2022-02-28

**Authors:** Hazel Quek, Carla Cuní-López, Romal Stewart, Tiziana Colletti, Antonietta Notaro, Tam Hong Nguyen, Yifan Sun, Christine C. Guo, Michelle K. Lupton, Tara L. Roberts, Yi Chieh Lim, Lotta E. Oikari, Vincenzo La Bella, Anthony R. White

**Affiliations:** 1 QIMR Berghofer Medical Research Institute, Brisbane, QLD Australia; 2 ALS Clinical Research Centre and Laboratory of Neurochemistry, Department of Experimental Biomedicine and Clinical Neurosciences, University of Palermo, Palermo, Italy; 3 Ingham Institute for Applied Medical Research and School of Medicine, Western Sydney University, Liverpool, NSW Australia; 4 Brain Tumour Biology, Danish Cancer Society, Copenhagen, Denmark

**Keywords:** Amyotrophic lateral sclerosis, Microglia, TDP-43 inclusions, DNA damage, Inflammasome

## Abstract

**Background:**

Amyotrophic lateral sclerosis (ALS) is a multifactorial neurodegenerative disease characterised by the loss of upper and lower motor neurons. Increasing evidence indicates that neuroinflammation mediated by microglia contributes to ALS pathogenesis. This microglial activation is evident in post-mortem brain tissues and neuroimaging data from patients with ALS. However, the role of microglia in the pathogenesis and progression of amyotrophic lateral sclerosis remains unclear, partly due to the lack of a model system that is able to faithfully recapitulate the clinical pathology of ALS. To address this shortcoming, we describe an approach that generates monocyte-derived microglia-like cells that are capable of expressing molecular markers, and functional characteristics similar to in vivo human brain microglia.

**Methods:**

In this study, we have established monocyte-derived microglia-like cells from 30 sporadic patients with ALS, including 15 patients with slow disease progression, 6 with intermediate progression, and 9 with rapid progression, together with 20 non-affected healthy controls.

**Results:**

We demonstrate that patient monocyte-derived microglia-like cells recapitulate canonical pathological features of ALS including non-phosphorylated and phosphorylated-TDP-43-positive inclusions. Moreover, ALS microglia-like cells showed significantly impaired phagocytosis, altered cytokine profiles, and abnormal morphologies consistent with a neuroinflammatory phenotype. Interestingly, all ALS microglia-like cells showed abnormal phagocytosis consistent with the progression of the disease. In-depth analysis of ALS microglia-like cells from the rapid disease progression cohort revealed significantly altered cell-specific variation in phagocytic function. In addition, DNA damage and NOD-leucine rich repeat and pyrin containing protein 3 (NLRP3) inflammasome activity were also elevated in ALS patient monocyte-derived microglia-like cells, indicating a potential new pathway involved in driving disease progression.

**Conclusions:**

Taken together, our work demonstrates that the monocyte-derived microglia-like cell model recapitulates disease-specific hallmarks and characteristics that substantiate patient heterogeneity associated with disease subgroups. Thus, monocyte-derived microglia-like cells are highly applicable to monitor disease progression and can be applied as a functional readout in clinical trials for anti-neuroinflammatory agents, providing a basis for personalised treatment for patients with ALS.

**Supplementary Information:**

The online version contains supplementary material available at 10.1186/s12974-022-02421-1.

## Introduction

Amyotrophic lateral sclerosis (ALS) is a debilitating disease characterised by the loss of motor neurons in the brain and spinal cord, resulting in progressive muscle weakness and eventual death. 90% of all ALS cases are sporadic (SALS) without a family history, while 10% are due to inherited genetic mutations (familial ALS) [1]. Moreover, mutations within common ALS-associated genes such as *SOD1* and *TARDBP* account for only 2% of ALS cases [2]. However, the majority of ALS patients exhibit aberrant cytoplasmic TAR DNA-binding protein-43 kDa (TDP-43) inclusions (from the *TARDBP* gene) in neurons and glial cells within CNS, predominantly in the motor cortex and spinal cord [3–7]. At present, there is no known cure and few effective treatments available for ALS, which is attributed to the multifactorial characteristics of this disease, including genetic susceptibility [8], clinical heterogeneity [9] and environmental exposure [10].

Accumulating evidence suggests that the death of motor neurons in ALS is a non-cell-autonomous event, which is likely exacerbated by non-neuronal neuroinflammatory responses [11, 12] primarily driven by microglia and astrocyte activation, followed by infiltrating peripheral immune cells such as monocytes and T lymphocytes [13, 14]. Neuroimaging studies have demonstrated increased neuroinflammation in ALS patients' primary and supplementary motor cortex and the prefrontal cortex [15–17]. Moreover, post-mortem ALS brain [18, 19] and spinal cord [20–24] tissues have also revealed substantial microglial pathology along with immune cell infiltrations. In the past, microglia activation was assumed to occur due to neuronal death rather than initiating the onset or progression of ALS. However, recent reports have shown extensive microglia activation prior to motor neuron loss in mouse models of ALS [25, 26].

Microglia appear to display a neuroprotective role in an early stage of the disease but progresses to a neurotoxic role later, subsequently accelerating motor neuron cell death [27–31]. Hence, the multifaceted role of microglia, implicated in ALS and other neurodegenerative diseases, makes these cells ideal candidates for developing therapeutic targets. However, it remains challenging to delineate precisely how microglia contribute to the different stages of ALS, especially in a patient-specific manner. Therefore, current therapeutic strategies that suppress microglial activation in a one-size-fits-all strategy could be ineffective [11, 32]. Indeed, only a few studies were able to correlate microglial pathology to genetic or clinical phenotypes in patients with ALS [15, 20, 33]. Hence a cellular model that can recapitulate the clinical heterogeneity in patients with ALS is urgently required.

In the past, research carried out to investigate the involvement of microglia in ALS relied predominantly on animal models and post-mortem tissue from the human central nervous system (CNS). However, the sampling of microglia from human brain autopsy and biopsy tissue is not practical for high-throughput drug screening platforms. Moreover, culturing isolated human microglia ex vivo is challenging due to its restricted proliferative capacity, cell viability, and rapid changes to its unique CNS identity once removed from the brain microenvironment [34]. Recent approaches using induced pluripotent stem cell (iPSC)-derived microglia are now available but have yet to provide significant insights into ALS neuroinflammatory processes, likely due to the relative complexity, high variability and increased time-frames required to generate iPSC-derived microglia. Moreover, iPSC-derived microglia may not accurately recapitulate the heterogeneity of clinical features observed in this disease due to the loss of epigenetic factors during the reprogramming of skin cells to stem cells [35].

To address these limitations and provide further insights into the pathological role of microglia in ALS, we generated and characterised patient-derived microglia-like cells from peripheral blood-derived monocytes [36, 37]. The monocyte-derived microglia-like cell (MDMi) model is a rapid, minimally invasive system that allows for multiple sampling at various stages of the disease and recapitulates the changes in microglia during the progression of ALS, thereby providing better clinical outcomes.

## Materials and methods

### Patient recruitment

30 sporadic ALS patients, including 15 patients with slow progression, 6 with intermediate progression, and 9 with rapid progression, were recruited from the ALS clinical Research Centre in Palermo, Italy. Healthy control (HC) were recruited from either the ALS Clinical Research Centre or the *Prospective Imaging Studying of Aging: Genes, Brain and Behaviour* study (PISA) at QIMR Berghofer Medical Research Institute, Queensland, Australia. The recruitment of HC participants was carried out as previously described in [38]. Briefly, recruitment/inclusion criteria of healthy research participants were within the age range of 40–80 years, fluent in English, and able to provide informed consent. Exclusion criteria of research participants included the presence of neurological disorders, history of neurosurgery, medical conditions that may confound neuropsychological testing, alcohol abuse, females who are pregnant or breastfeeding, history of severe psychiatric illness, presence of prostheses [38]. All research adhered to the human research ethical guidelines outlined by the National Health and Medical Research Council of Australia (NHMRC), the QIMR Berghofer Medical Research Institute, and the University of Palermo. All participants provided informed consent before participating in the study.

Patients with ALS were diagnosed according to the El-Escorial criteria, with a balanced proportion of bulbar and spinal onset. All patients provided detailed family history and were tested for common genetic mutations (SOD1, C9orf72, etc.). Only one patient was a carrier of sextuplets GGGGCC C9orf72 with a > 30 sextuplets expansion. The degree of functional impairment was assessed with the Revised Amyotrophic Lateral Sclerosis Functional Rating Scale (ALSFRS-R) [39]. The rate of disease progression was evaluated using the ALSFRS-R score: progression rate ratio (Delta FS, ΔFS) according to the following formula: (48—ALSFRS-R score at the time of diagnosis)/time onset to diagnosis). Patients were then stratified into three arbitrary groups according to ΔFS: slow (ΔFS < 0.5), intermediate (ΔFS = 0.5–1.0), rapid (ΔFS > 1.0), which can predict life expectancy post diagnosis [40, 41]. Disability, as calculated by ALSFRS-R at entry, was mild in the whole cohort, but became worst in the rapid progressors. All patients were Caucasian, and were recruited from a single ALS Centre in Palermo, Sicily. A summary of disease characteristics, and demographics of all patients and control donors used in this study, are shown in Table 1.

**Table 1:** Summary of donor information

Study Cohorts:	Healthy Control	ALS			*P *value
Disease progression		Slow	Intermediate	Rapid	
No. of participants	*N* = 20	*N* = 15	*N* = 6	*N* = 9	
Sex of participants					
Females	55% (11/20)	33.3% (5/15)	66.7% (4/6)	44.4% (4/9)	0.46^c+^
%, (proportion)					
Males	45% (9/20)	66.7% (10/15)	33.3% (2/6)	55.6% (5/9)	
%, (proportion)					
Age: Mean (± SD)	65.3 (8.9)	57.2 (10.2)	70.3 (9.7)	66.0 (8.84)	0.40^d+^
Site of onset	–				
Bulbar: %, (proportion)		13.3% (2)	16.7% (1)	22.2% (2)	0.85^c−^
Spinal: %, (proportion)		86.7% (13)	83.3% (5)	77.8% (7)	
ALSFRS-R^a^: mean, ± SD (range)		41.6, ± 3.4 (34–46)	41, ± 2.3 (38–44)	36.8, ± 5.04 (30–46)	0.02^d−^
ΔFS^b^: mean, ± SD (range)		0.25, ± 0.1 (0.1–0.45)	0.60, ± 0.1 (0.57–0.67)	1.72, 0.76 (1–3.4)	0.001^d−^

^a^ALSFRS-R (at diagnosis, when PBMCs were taken; normal score = 48)

^b^Delta FS ratio (rate of disease progression); (48- ALSFRS-R score at time of diagnosis)/ time onset to diagnosis)

*SD* Standard deviation

*ALSFRS-R* Revised Amyotrophic Lateral Sclerosis Functional Rating Scale;

^c^Chi-square test

^d^ANOVA

^+^*p* value for comparison between HC and ALS cohort

^−^*p* value for comparison between ALS subgroups

The number of samples varied for each assay due to the limited proliferative capacity of MDMi in culture and the number of blood samples available from each patient. Moreover, repeated longitudinal sampling of peripheral blood from patients was not within the scope of this study. All samples used for assays were randomly selected and matched for age and gender (listed in the respective figure legends).

### Isolation of PBMCs from blood samples

Donor blood was collected into K_3_ ethylenediaminetetraacetic acid (EDTA) tubes diluted 1:1 with phosphate-buffered saline (PBS) and transferred into sterile SepMate50 (STEMCELL Technologies, CA) and centrifuged at room temperature at 1200 g for 30 min at room temperature according to the manufacturer's guidelines. Plasma was then removed by aspiration, and Peripheral blood mononuclear cells (PBMCs) were transferred into a 50 ml conical tube and resuspended with sterile PBS. The tube was then centrifuged at room temperature at 400 g for 10 min, after which the supernatant was removed by aspiration and the PBS wash step was repeated. The PBMC pellet was then resuspended in freezing media containing 90% v/v fetal bovine serum (FBS) and 10% dimethyl sulfoxide (Thermofisher, USA) and transferred into 1.8 ml Nunc CryoTubes cryogenic vials (Thermofisher, USA). The vials were placed overnight at − 80 °C before cryopreservation in liquid nitrogen.

### Generation of monocyte-derived microglia-like cells (MDMi) from PBMCs

Cryopreserved PBMCs were thawed into 9 ml of pre-warmed RPMI-1640 GlutaMax media (Life Technologies, USA) supplemented with 10% heat-inactivated FBS (Life Technologies, USA) and centrifuged at room temperature for 5 min at 300 *g*. The supernatant was then aspirated completely, and the cell pellet was resuspended in RPMI-1640 GlutaMax media (Life Technologies, USA) supplemented with 1% penicillin–streptomycin (P/S) and 10% FBS. According to the manufacturer's instructions, cells were then plated onto Matrigel-coated plates (8.7 µg/cm^2^ in PBS) using the thin coating method. For differentiation of PBMCs into MDMi, RPMI-1640 GlutaMax media was supplemented with 0.1 µg/ml of interleukin (IL)-34 (Lonza, CH) and 0.01 µg/ml of granulocyte–macrophage colony-stimulating factor (GM-CSF) (Lonza, CH). Cells were cultured for 7 or 14 days depending on experimental aims, with media changes performed every third day. On day 13, conditioned media was collected and replaced with fresh medium. This conditioned media was centrifuged for 5 min at 400 *g* and stored at − 80 °C for downstream multiplex bead-based immunoassays. Cultures of MDMi were harvested for downstream experiments on day 7 or 14.

Differentiation of PBMCs into MDMi was confirmed by 1) presence of the classical ramified morphology, 2) immunofluorescence for microglial markers, and 3) increased mRNA expression of microglial specific genes in comparison to monocyte-derived macrophages (MDMa) and monocyte-derived dendritic cells (MDDCs). All experiments were performed on a batch-to-batch basis. Each batch of experiments consisted of unique PBMCs for differentiation. To maximise sample usage from each patient and increase the number of patient-control replicates for each experiment, ALS PBMCs were matched with appropriate HC PBMCs.

Monocytes were isolated from PBMCs according to the methodology described by several groups [36, 37, 42–45]. Briefly, PBMCs were cultured overnight on Matrigel-coated plates. Unbound suspension cells were removed, and the wells were washed thoroughly with RPMI-1640 GlutaMax media before collecting for downstream experiments.

### Generation of monocyte-derived macrophages (MDMa) and dendritic cells (MDDCs) from PBMCs

For MDMa induction, PBMCs were seeded in 48-well plates without prior coating with Matrigel in RPMI-1640 GlutaMax media supplemented with 10% heat-inactivated FBS and 10 ng/ml GM-CSF and cultured for 7 or 14 days, with media changes performed every third day. MDMa cultured in this manner displayed typical macrophage features such as a large cell surface area and amoeboid shape, distinct from the morphological features of MDMi (Additional file 1: Fig. S1b).

For MDDC induction, PBMCs were seeded in 6-well plates without prior coating with Matrigel in RPMI-1640 GlutaMax media supplemented with 10% heat-inactivated FBS and 50 ng/ml of IL-4, and 100 ng/ml of GM-CSF. Cells were cultured for 7 days and either harvested for downstream assays or induced with 100 ng/ml lipopolysaccharide (LPS, O55:B5, Sigma-Aldrich, USA) and 1000 IU/ml IFNγ (Lonza, CH) for 48 h for MDDC maturation [46]. Mature MDDCs displayed a characteristic stellate morphology (multiple pseudopodia), distinct from MDMa and MDMi (Additional file 1: Fig. S1b).

### Culture conditions of ReNcell VM cell line

The human ReNcell VM immortalised neural progenitor cell line (EMD Millipore, USA) was cultured as per the manufacturer’s guidelines. Briefly, 2 × 10^6^ cells were plated onto Matrigel-coated T75 cell culture flasks and maintained in DMEM/F12 GlutaMax medium (Thermofisher, USA) containing 2% (v/v) B27 supplement, 20 µg/ml epithelial growth factor (EGF) (Lonza, CH), 20 µg/ml fibroblast growth factor 2 (FGF-2) (Lonza, CH) and 1% (v/v) penicillin/streptomycin with media changes performed every 3 days. ReNcell VM cells were detached with accutase solution (Thermofisher, USA) according to the manufacturer’s guidelines.

### Live cell imaging of phagocytosis

Characterisation of phagocytic function was performed via live-cell imaging on the IncuCyte ZOOM (Essen Biosciences). In brief, sonicated fluorescent pHrodo-labelled *E.coli* particles (Thermofisher, USA) were added to MDMi cultures. Multiple images were captured every hour at 10 × magnification for a total of 15 h using standard phase contrast and red fluorescence settings. Data were analysed using the IncuCyte ZOOM software 2018A. Background fluorescence was subtracted from original images using the top-Hat threshold transform algorithm. All parameters were kept constant throughout all experiments. Normalised phagocytosis was calculated as (Red object area)/ (Phase object count) for each time point, and quantifications were performed blinded until analyses were complete and patient details were declassified.

### RNA extraction and quantitative real-time polymerase chain reaction (qRT-PCR)

Total RNA extraction was performed using Direct-zol RNA miniprep kit (Integrated Sciences, AUS) according to the manufacturer's instructions as previously described [47]. cDNA synthesis was then performed using a SensiFast cDNA synthesis kit (Bioline, UK). Samples were diluted and mixed with SensiFAST Sybr Lo-Rox master mix before loading as triplicates for qRT-PCR. qRT-PCR was performed using the Applied Biosystems ViiA 7 system. Similar *18S* ribosomal RNA expression was observed in HC and in ALS MDMi. Delta cycle threshold (ΔCT) values were normalised to *18S* and used to determine relative gene expression between samples. Samples with CT values more than two times the standard deviation of the average of each reference gene were excluded from the analysis. Melting curve analyses confirmed a single melting curve peak for all primers. The primers used in this study are summarised in Additional file 1: Table S1.

### Immunofluorescence and confocal microscopy analysis

Cells were plated onto 8-well chamber slides (Ibidi), and immunofluorescence was performed as previously described in [48]. Briefly, cells were fixed with 4% paraformaldehyde in PBS. Permeabilisation of PFA fixed samples was performed with PBS containing 0.3% Triton-X 100 (Sigma-Aldrich, USA). Samples were blocked with PBS containing 5% bovine serum albumin (BSA) (Sigma-Aldrich, USA). Primary antibodies [Anti-P2RY12 (Alomone Labs, #APR-20, 1:200), Anti-IBA1 (Abcam, #Ab5076, 1:500; Wako, #019-19741, 1:500), Anti-TDP-43 (Cosmo Bio, #TIP-TD-P09, 1/500), Anti-p-TDP43 (Cosmo Bio, #TIP-TD-P09, 1:500), Anti- γH2AX (EMD Millipore, #05-636, 1:400), Anti-ASC (AdipoGen, #AG-25B-0006-C100, 1:200), Anti-NLRP3 (AdipoGen, #AG-20B-0014-C100, 1:500)] were incubated overnight. Cells were washed thrice with 0.1% Triton-X in PBS followed by incubation with secondary antibodies and DAPI (nuclear dye) (Sigma-Aldrich, USA) for 2 h at room temperature. Antibody specificity was confirmed by performing secondary antibody only controls. Investigators were blinded to the conditions of the experiments during data collection and analyses. Images were captured with a confocal laser scanning microscope (LSM-780, Carl Zeiss) with all settings kept consistent during acquisition. Colocalisation between NLRP3 and ASC was analysed using ImageJ software (ImageJ 1.52n, National Institutes of Health, Maryland, USA), using the plugin "Coloc2". Merged images were split into individual channels, and a background correction was applied. Cell masks were determined, constructed and added to "ROI manager" according to IBA-1 staining within cells. The summarised colocalisation efficiency data was expressed as Pearson's correlation coefficiency (PCC). As previously described, the PCC correlates intensity between signals of two probes [49]. 10 cells per individual were analysed, and a summary of the PCC data for each cohort was used for statistical analysis.

### Multiplex bead-based immunoassay for cytokine and chemokine

Unstimulated HC and ALS MDMi conditioned media was harvested on day 13, centrifuged at 400 *g* for 5 min to remove cell debris and stored at − 80 °C until use. Cytokine and chemokine concentrations were measured using a bead-based multiplex LEGENDplex™ Human Inflammation Panel 1 (13-plex, BioLegend, USA) kit as per the manufacturer's instructions. The panel of 13 cytokine/chemokines included IL-1β, IFN-α2, IFN-γ, TNF-α, MCP-1 (CCL-2), IL-6, IL-8 (CXCL8), IL-10, IL-12p70, IL-17A, IL-18, IL-23, and IL-33. Sample acquisition was performed in duplicates using a flow cytometer, and the data were analysed using Qognit, a cloud-based software (BioLegend, USA) specified at pg/ml values. Cytokines below the limit of detection (LOD) were excluded from the analysis (including IL-17A and IL-12p70).

### TDP-43 treatment

Human recombinant TDP-43/TARDBP protein (R&D Systems, USA) was reconstituted as per the manufacturer's guidelines. 1 and 10 nM of the reconstituted protein were added to 14-day-old MDMi cultures for 24 h prior to harvesting.

### Skeleton analysis of MDMi morphology

Phase-contrast images of MDMi were captured with a spinning disc confocal microscope using a 20 × objective. The characterisation of microglial morphology was performed using ImageJ analysis software. Phase-contrast images of MDMi were first pre-processed using a macro script that applied a threshold, followed by conversion to binary images. The "AnalyzeSkeleton" plugin was then executed on the binarised images to analyse MDMi branch length, branch number (cell process and end-points per cell), and branch junctions (triple or quadruple junctions). These data measure microglial morphology (complexity and process length) [50]. 100 cells per individual in each subgroup were analysed.

### Quantification of nuclear-to-cytoplasmic TDP-43

The nuclear-to-cytoplasmic ratio was analysed using Image J analysis software using methodology adapted from [51]. Briefly, images were first pre-processed using a macro script that applied a background subtraction followed by threshold, then finally a conversion to binary image. Images were then processed using tools such as "Dilate" or "Fill holes" before "Analyse particle" was performed. IBA-1 and DAPI stained cell images were used to create a mask for whole-cell and nucleus, respectively. Determination of nuclear TDP-43 was performed using a nuclear mask generated using DAPI [added to ROI manager and superimposed (by "show all" in ROI manager)] on images stained only for TDP-43. A similar strategy was applied for generating a whole-cell mask. Integrated density (Area X Mean Gray Values) was measured using the appropriate mask for the whole cell and the nucleus. The cytoplasmic intensity was calculated by: (whole-cell integrated density – nuclear-integrated density). Finally, the nuclear-to-cytoplasmic ratio of TDP-43 was calculated by nuclear-integrated density / cytoplasmic intensity.

### Statistical analysis

Data are presented as either mean ± SD or SEM. Normality was accessed by Shapiro–Wilk normality tests. For normal distribution, comparisons between ALS and HC cohorts were performed using the Student's *t* test (two-tailed) while multiple comparisons were performed using ordinary one-way ANOVA (to compare multiple treatment groups versus control). For non-normal distribution, the Mann–Whitney *U* test (two-tailed) was used to compare ALS and the HC cohort. For non-normal multiple comparisons, a Kruskal–Wallis one-way ANOVA (non-parametric) followed by Dunn's multiple comparisons test performed (to compare multiple treatment groups versus controls). Correlations were analysed by Spearman rank correlation test. All statistical analyses were performed using GraphPad Prism 9 (Graphpad Software). *P < 0.05 was considered statistically significant.

## Results

### Generation of human monocyte-derived microglia-like cells (MDMi)

**Fig. 1:**
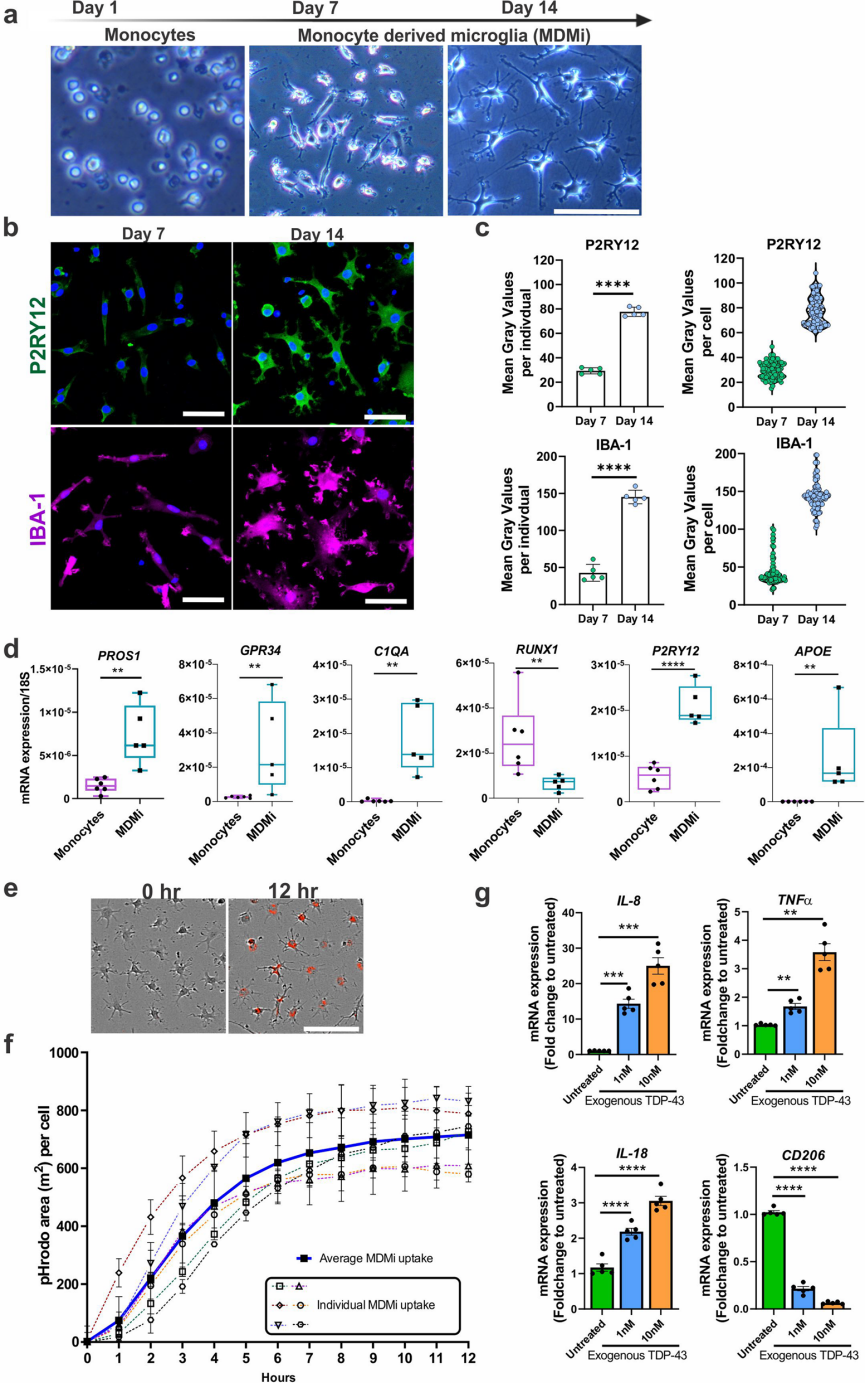
Generation and characterisation of human monocyte-derived microglia-like cells (MDMi).** a** Schematic timeline of MDMi differentiated for 1, 7 and 14 days in culture, with representative phase contrast images. **b** Immunofluorescence images of P2RY12, IBA1, with counter-stain DAPI in MDMi cultured on Day 7 and Day 14 (*n* = 5). **c** MDMi have higher P2RY12 and IBA1 protein levels at Day 14 compared to Day 7 as measured for 5 different individuals (*n* = 100 cells in total). **d** Gene expression of seminal microglia genes including *PROS1, GPR34, C1QA, RUNX1, P2RY12* and *APOE* between isolated monocytes (*n* = 6) and MDMi (Day 14, *n* = 5). **e** Representative phase contrast images of day 14 MDMi with pHrodo-labelled *E.coli* particles (red) uptake at 12 h after treatment compared to untreated (left panel) (*n* = 6). **f** Quantification of phagocytosis by pHrodo-labelled *E.coli* particles over 12 h using live imaging in healthy volunteers (n = 6, dotted lines). Average uptake of MDMi shown in bold line. **g** Gene expression of MDMi (Day14) treated with 1 nM (blue) and 10 nM (orange) recombinant TDP-43 protein (*n* = 5). The *y*-axis represents the fold change of mRNA expression levels (*IL-8, TNFα, IL-18* and *CD206*) normalised to untreated cells over 24 h treatment. Data were first tested for normality using Shapiro–Wilk test. Statistical analysis between two groups was performed using Student’s *t* test and between multiple groups using one-way ANOVA. Values are the mean ± SD (*P < 0.05, **P < 0.01, ***P < 0.001, ****P < 0.0001). Scale bars = 50 µm

Initial characterisation of MDMi was performed with PBMCs collected from healthy volunteers. Isolated PBMCs were cultured for 14 days with induction media consisting of IL-34 (100 ng/ml) and GM-CSF (10 ng/ml) as previously described [36, 37], with some minor modifications (Additional file 1: Table S2). MDMi cultured for 14 days showed microglia-like characteristics such as a small soma with ramified morphology comparable to resting-state microglia and positive immunostaining for markers enriched in microglia such as P2RY12 and IBA1 compared to monocytes (Fig. 1a, b). Additionally, MDMi cultured for 14 days displayed higher P2RY12 and IBA1 protein levels than MDMi cultured for 7 days (Fig. 1c). We then examined seminal microglial markers selected from several transcriptome studies [37, 52–54] and observed an overall increase in the expression levels of *PROS1, GPR34, C1QA, MERTK, GAS6, APOE* and *P2RY12* [37, 52–54] (Fig. 1d), and commonly described microglial genes such as *TREM2, CD68,* and *HLA-DRA* in MDMi at day 14 compared to monocytes (Additional file 1: Fig. S1a). *RUNX1*, a key regulator of myeloid proliferation and differentiation, is downregulated in ramified microglia [55, 56]. Consistent with published studies, we observed a clear decrease in *RUNX1* expression in MDMi compared to monocytes. However, there was no change in the expression levels of transcription factors that have an essential role in regulating both monocytes and microglia, such as *PU.1* and *IRF8*, at day 14, which further validates our results [57, 58].

Additionally, we compared the morphology and mRNA expression of MDMi to monocyte-derived macrophages (MDMa), and monocyte-derived dendritic cells (MDDCs) (Additional file 1: Fig. S1b). Compared to MDMi, MDMa displayed typical amoeboid morphology with decreased expression of microglial-markers, *TREM2*, *CX3CR1* and *CD68*, and an increased expression of the pan-leukocyte marker, *CD45* (Additional file 1: Fig. S1c). Moreover, compared to MDDCs, MDMi expresses CD209, an immature MDDC marker and CCR7, a mature MDDC marker, at very low levels, but expressed CD68, a microglial marker, at a higher level (Additional file 1: Fig. S1d). Together these results confirm the identity of our MDMi population and show that they are distinct from MDMa and MDDCs.

Next, we validated the functional capacity of MDMi cells by stimulating MDMi with pHrodo-labelled *E.coli* particles, where uptake into the acidic phagosomes results in fluorescence. An increased in red intensity over time confirmed the phagocytic ability of MDMi (Fig. 1e, f and Additional file 2: Video S1). We then further examined the immune response of MDMi to transactive response DNA-binding protein-43 (TDP-43), a pathogenic protein involved in 95% of all sporadic ALS cases [59]. Interestingly, MDMi treated with 1 nM or 10 nM of recombinant TDP-43 protein for 24 h showed a dose-dependent increase of pro-inflammatory cytokines (*IL-8*, *TNFα* and *IL-18*) and a decrease in the anti-inflammatory cell surface marker (*CD206*) compared to untreated MDMi (Fig. 1g). Together, these results confirm that MDMi are functional and are capable of recapitulating specific tasks performed by brain microglia in vivo.

### Altered microglial morphology in ALS MDMi

**Fig. 2:**
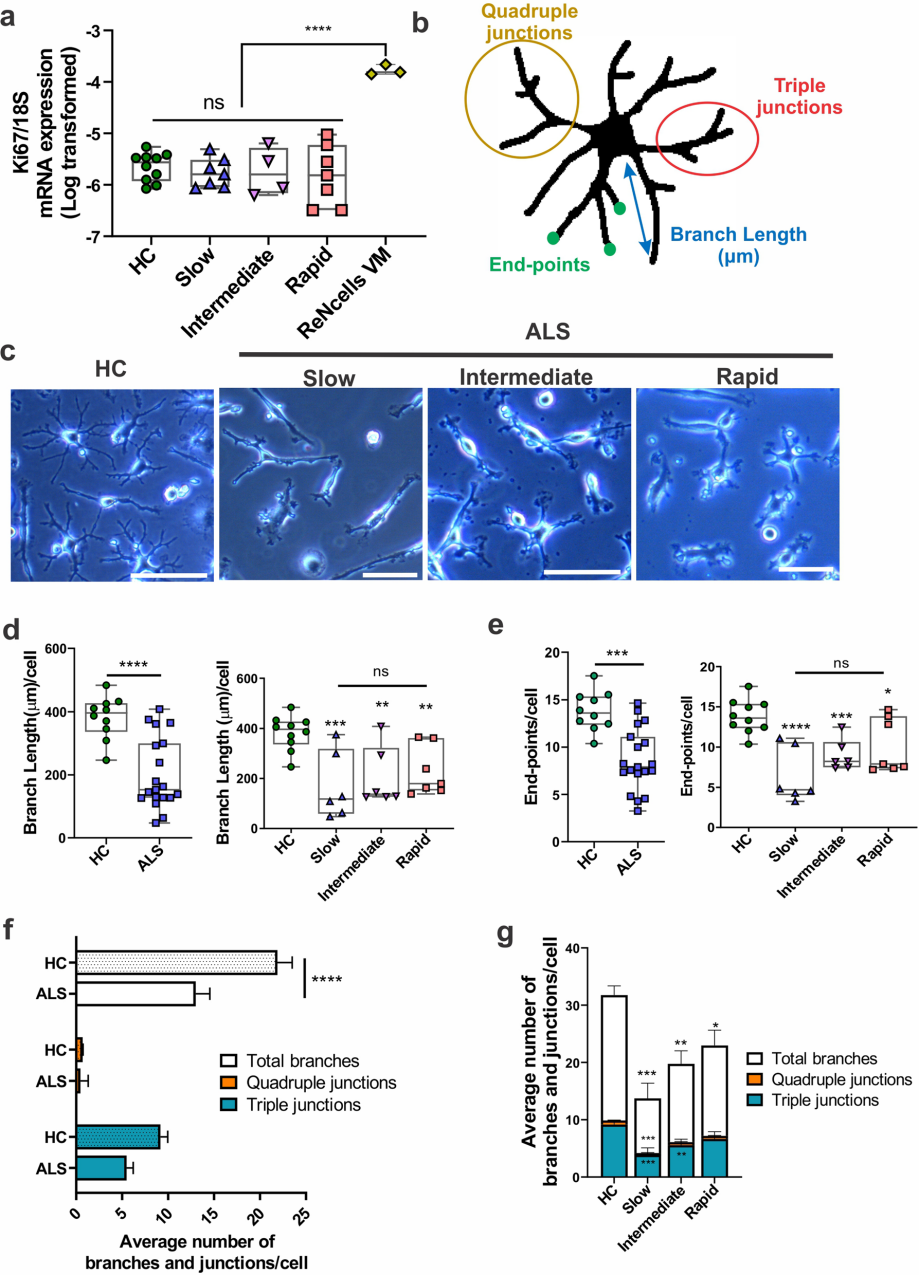
Morphology of ALS MDMi compared to HC MDMi** a** Log transformed gene expression of *Ki67* mRNA normalised to housekeeping gene, *18S*, was examined in HC, slow, intermediate, and rapid ALS MDMi subgroups at day 14. A commercially available neural stem cell line (ReNcell VM) was used as a control for proliferating cells, *n* = 3 from independent experiments. No differences were observed between age-matched HC and ALS cohort or between ALS MDMi subgroups; HC: *n* = 10, Slow: *n* = 7, Intermediate: *n* = 4, Rapid: n = 7. **b** Schematic example of a skeletonised MDMi showing branch length (µm), end-points, triple junctions (junctions with exactly 3 branches), and quadruple junctions (junctions with exactly 4 branches). **c** Phase contrast images of mature ALS MDMi subgroups (slow, intermediate, and rapid) 14 days in culture. **d** Microglial branch length (left panel) was compared between the ALS (*n* = 19) and HC (*n* = 10) MDMi cohorts on day 14. The ALS MDMi cohort was then categorised into slow, intermediate and rapid subgroups for microglial branch length (right panel). **e** Microglial end-points (left panel) were compared between ALS (*n* = 19) and HC (*n* = 10) MDMi cohort on day 14. The ALS MDMi cohort were then categorised into slow, intermediate and rapid subgroups for microglial end-points (right panel). Both microglial branch length and end-points were analysed by ImageJ and normalised against cell number. HC: *n* = 10, ALS: *n* = 19 (Slow: *n* = 6, Intermediate: *n* = 6, Rapid: *n* = 7). **f** Average number of branches, and secondary junctions of HC and ALS MDMi and **g** ALS MDMi subgroups were analysed by ImageJ and normalised against cell number; HC: *n* = 10, ALS: n = 19 (Slow: *n* = 6, Intermediate: *n* = 6, Rapid: *n* = 7). Data were first tested for normality using Shapiro–Wilk test. Statistical analysis between two groups was performed using Student’s *t* test and between multiple groups using one-way ANOVA. Values are the mean ± SD (in Figures a, d and e) and SEM (in Figures f and g) (*P < 0.05, **P < 0.01, ***P < 0.001, ****P < 0.0001). Scale bars = 50 µm

As microglia mature, they lose their ability to proliferate [60]. Hence we next investigated if in vitro MDMi resemble mature microglia by determining their proliferative capacity using *Ki67* expression, a cell proliferation marker. In addition, as a positive control for cell proliferation in each of these experiments, we used the commercially available ReNcell VM cell line. Interestingly, similar expression levels of *Ki67* was observed across HC and ALS disease subgroups, confirming the presence of mature differentiated MDMi in all cohorts (Fig. 2a).

Microglial activation in vivo is accompanied by changes in morphology, where microglia in an activated state switch from a ramified to an amoeboid-like morphology [61]. To examine morphology changes in ALS MDMi, we measured branch length, end-points and branch junctions (Fig. 2b, c, Additional file 1: Fig. S2a).

An overall significant reduction in branch length (*P* < 0.0001), and end-points (*P* < 0.0001) were observed in ALS compared to age-matched HCs MDMi (Fig. 2d, e, left panels). Next, we stratified ALS MDMi into three different rates of disease progression (slow, intermediate and rapid) and observed a significant branch length reduction in all ALS subgroups, and end-points compared to HC MDMi (Fig. 2d, e, right panels), resulting in a decreased cell ramification in this cohort. However, a portion of ALS MDMi within each subgroup presented a similar morphology to HC MDMi, suggesting heterogeneity of cell morphologies within each disease subgroup due to patient-specific variability.

Additionally, we found a significant decrease in the total number of branches [including triple (*P* = 0.0008) and quadruple junctions (*P* = 0.0002)] per cell in ALS compared to HC MDMi, indicating that ALS MDMi are impaired in their ability to generate complex branches (Fig. 2f).

This reduction in overall branches and junctions were observed in all ALS disease subgroup compared to HC MDMi. Interestingly, we also found a reduction in the number of triple and quadruple junctions in the slow disease subgroup and a reduction in the number of triple junctions in the intermediate subgroup in ALS MDMi compared to HC. However, no differences in triple and quadruple junctions were observed in the rapid disease subgroup compared to HC MDMi (Fig. 2g). Overall, these results demonstrate that changes in MDMi morphology may be associated with disease progression, where the slow subgroup appears to be the least ramified, followed by both the intermediate and rapid subgroups.

### Abnormal cytoplasmic inclusions were positive for TDP-43 and/ or pTDP-43 in ALS MDMi

**Fig. 3:**
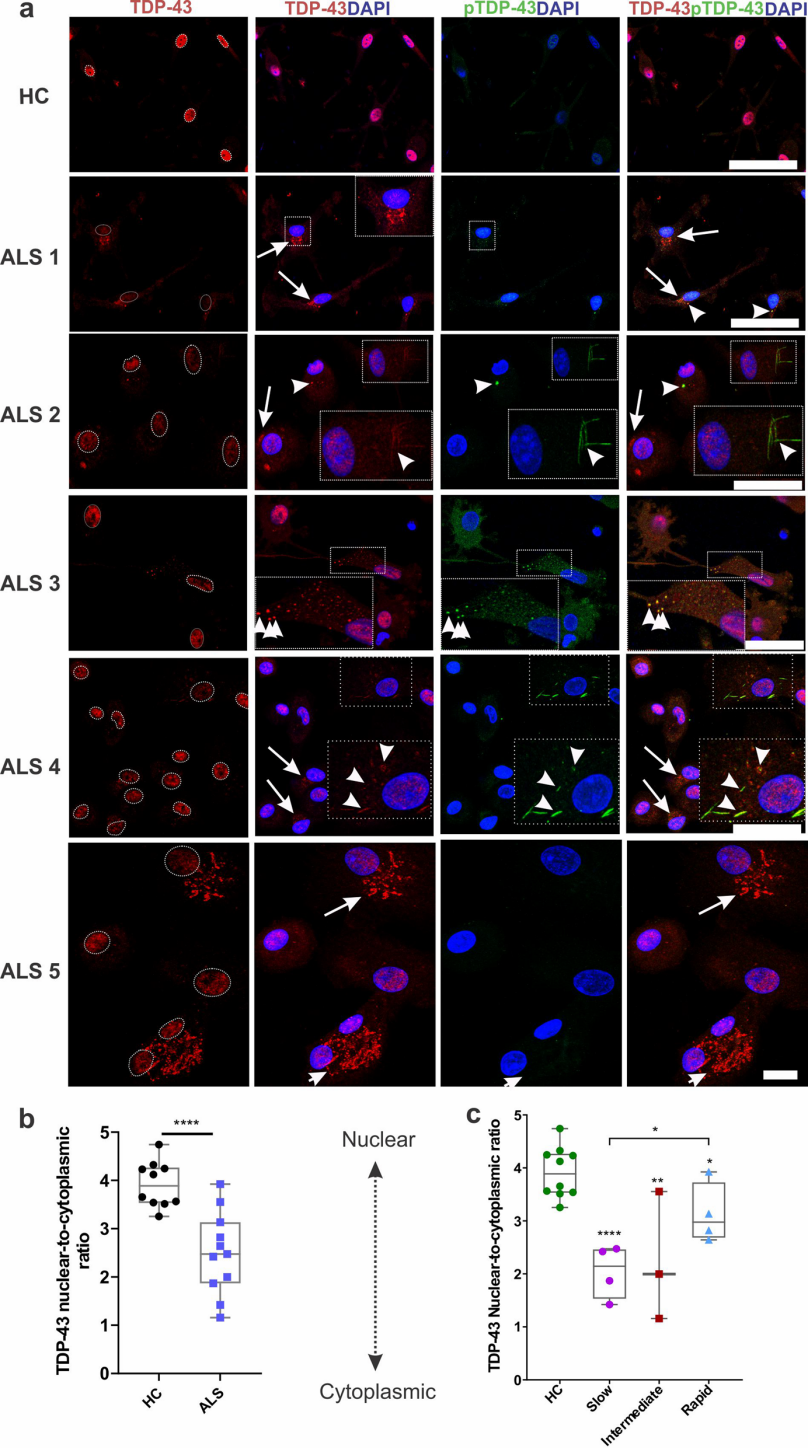
ALS MDMi display abnormal TDP-43 and/or pTDP-43 cytoplasmic localisation and inclusions** a** Representative immunofluorescence images of HC and ALS MDMi at day 14 showing TDP-43 (red), pTDP-43 (green), and DAPI counterstain (blue). Dotted circles in TDP-43 stained images (first column) represents the nucleus. White arrows indicate TDP-43 and/or pTDP-43 inclusions in ALS MDMi. Arrowheads indicate the co-localisation of TDP-43 and pTDP-43 inclusions. Inserts depict higher magnifications of the full images. **b** Ratio of nuclear-to-cytoplasmic TDP-43 in HC (*n* = 10) and ALS MDMi (*n* = 11) cohorts. **c** ALS MDMi from **b** was categorised into disease subgroups (Slow: *n* = 4, Intermediate: *n* = 3, Rapid: *n* = 4). Significantly increased cytoplasmic TDP-43 observed in slow, intermediate and rapid ALS cohorts compared to HC MDMi at day 14. n = 10 cells measured per individual MDMi. Data were first tested for normality using Shapiro–Wilk test. Statistical analysis between two groups was performed using Mann–Whitney *U* test and between multiple groups using Kruskal–Wallis test. Values are mean ± SD (*P < 0.05, **P < 0.01, ****P < 0.0001). Scale bars = 50 µm

A cellular hallmark of ALS is the accumulation of abnormal cytoplasmic TDP-43 aggregates in neurons, where TDP-43 depletes from the nucleus into the cytosol and forms toxic cytosolic inclusions, which accelerate disease progression [5, 62]. Given the toxic role of TDP-43 in neurons, we examined whether this TDP-43 pathology was also present in ALS MDMi. Immunofluorescence microscopy revealed a variety of cytoplasmic inclusions in ALS MDMi, including dashes, skein-like and round structures that stained positive for total TDP-43 (aa 405–414) (Fig. 3a, Additional file 1: Fig S3a). Interestingly, these abnormal TDP-43 pathological inclusions have also been observed in post-mortem brain and spinal cord tissues from ALS patients [3, 63, 64].

In addition, ALS MDMI showed a reduction of TDP-43 immunostaining in the nucleus and an increase of it within the cytoplasm (as either "punctate" or "granular" structures) [65] (Fig. 3a, ALS 1 and ALS 5). Interestingly, out of 11 ALS MDMi examined, we found 100% (4 out of 4 slow cases), 67% (2 out of 3 intermediate cases) and 50% (2 out of 4 rapid cases) that showed a reduction of TDP-43 in the nucleus and a corresponding increase in the cytoplasm. Conversely, 100% of the HC MDMi (10 out of 10) showed a strong nuclear TDP-43 immunostaining with no cytoplasmic component (Fig. 3b, c).

Another pathological hallmark of TDP-43 proteinopathy is the abnormal phosphorylation of TDP-43 inclusions, which drive the pathogenesis of ALS [66, 67]. Using an antibody against phosphorylated TDP-43 (pTDP-43 Ser409/410), we observed that a subset of pTDP-43 inclusions co-localised with TDP-43 inclusion (Fig. 3a. ALS 2, ALS 3, ALS 4, Additional file 1: Fig S3a). However, not all TDP-43 inclusions colocalised with the pTDP-43 inclusions (Fig. 3a, ALS 2 and ALS 5), suggesting that other post-translational modifications such as ubiquitination may be present within these structures.

Our results have demonstrated that TDP-43-positive cytoplasmic inclusions are heterogeneous and independent of the type of disease progression. Interestingly, TDP-43 cytoplasmic inclusion in ALS MDMi cultures formed under basal conditions without additional stressors, indicating a de novo TDP-43 pathology due to inherently elevated stress levels within these cells. Overall, our findings suggest that TDP-43 mislocalisation in MDMi may have a putative role in contributing to ALS pathology.

### Increased DNA damage and NLRP3 inflammasome formation in ALS MDMi

**Fig. 4:**
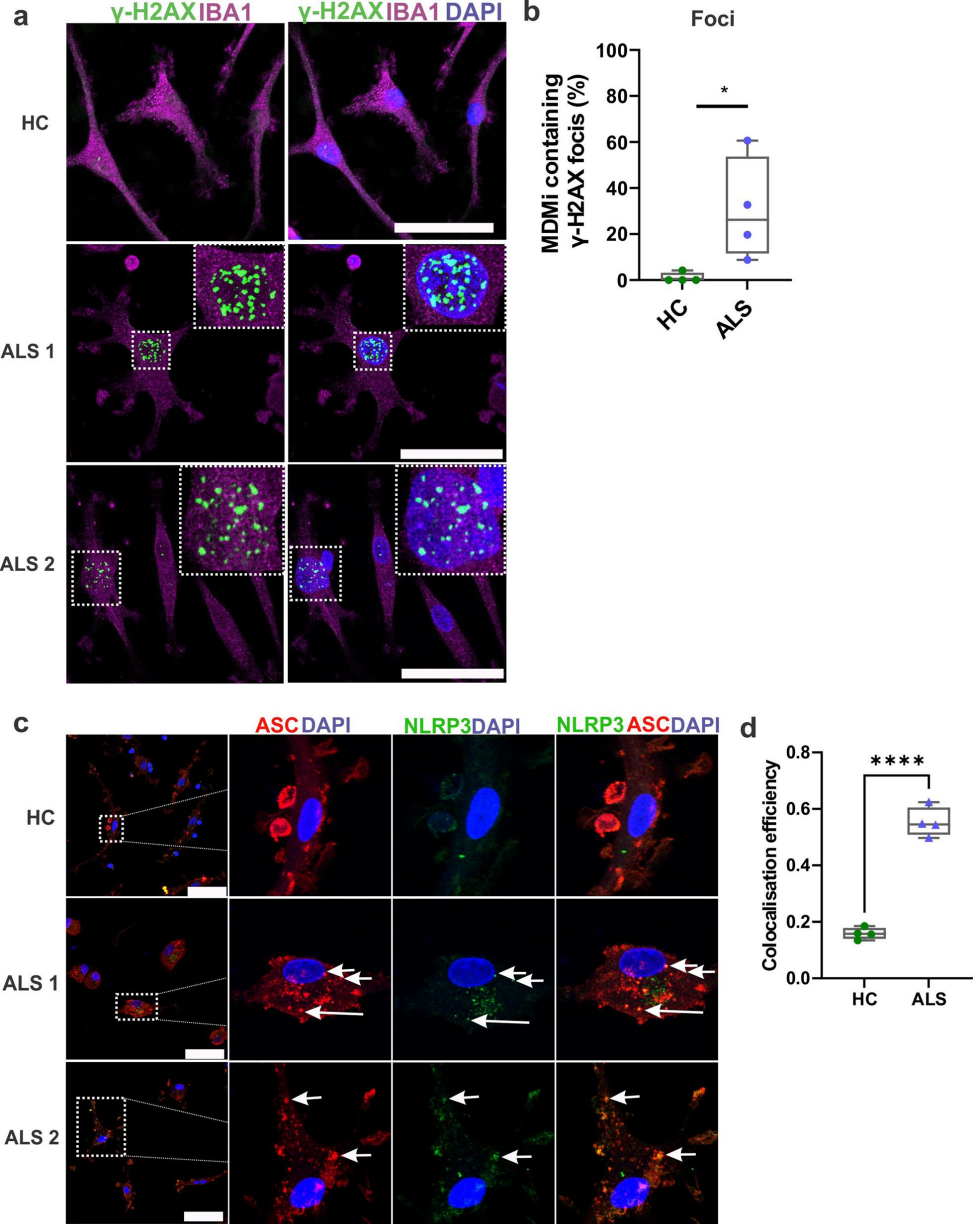
ALS MDMi display DNA damage and NLRP3 inflammasome formation.** a** Representative immunofluorescence images of ALS MDMi showing γH2AX (green), Iba1 (magenta), and DAPI counterstain (blue). Enlarged images on the top right indicates γH2AX foci in ALS MDMi. **b** Increased percentage of MDMi containing γH2AX foci in ALS (n = 4) compared to HC MDMi (*n* = 4, Day14). 60 cells per individual. Formula is as follows (number of cells containing γH2AX foci / total number of cells)*100. **c** Representative immunofluorescence images of HC and ALS MDMi showing NLRP3 (green), ASC (red) and DAPI counterstain (blue). NLRP3 in HC (top panel) does not co-localise with ASC, while ASC speck was co-localised with NLRP3 in ALS MDMi suggesting inflammasome formation. **d** Colocalisation (Pearson’s colocalisation efficiency) between NLRP3 and ASC in HC and ALS MDMi. HC: *n* = 4, ALS: *n* = 4, 10 cells per individual. All HC and ALS MDMi cultures were differentiated for 14 days and were unstimulated. Data were first tested for normality using Shapiro–Wilk test. Statistical analysis between two groups was performed using Student’s *t* test. ASC: apoptosis-associated speck-like protein containing a CARD; NLRP3: NLR family pyrin domain containing 3. Values are mean ± SD (*P < 0.05, ****P < 0.0001). Scale bars = 50 µm

The loss of nuclear TDP-43 in motor neurons has been linked to the accumulation of DNA damage within these cells [68]. Hence, we examined DNA damage using an antibody recognising phosphorylated H2AX (γH2AX), a marker for DNA double-strand breaks (DSBs). Interestingly, there was a significantly increased population of MDMi with γH2AX foci in ALS (30%) compared to HC (4%) (Fig. 4a, b). Moreover, we found that a subset of phosphorylated γH2AX positive ALS MDMi showed pan-nuclear staining, a characteristic that was absent in HC (Additional file 1: Fig. S3b, c). These pan-nuclear γH2AX structures are formed by clusters of γH2AX foci and are mechanistically and morphologically distinct from γH2AX foci [69].

The activation of NOD-leucine rich repeat and pyrin containing protein 3 (NLRP3) inflammasome complex has been observed in the microglia from mouse models of ALS [70, 71] and brain tissue samples from ALS patients [72, 73]. The assembly of the inflammasome complex involves the upregulation of the NLRP3 protein, the recruitment of ASC adapter protein, and caspase-1. This leads to the cleavage of pro-IL1 β and pro-IL-18 followed by the release of mature IL-1β and IL-18 cytokines and subsequent pyroptotic cell death [74]. When the inflammasome is activated, cytosolic ASC assembles into a singular, perinuclear, speck-like structure that colocalises to NLRP3 [75]. Microglial NLRP3 inflammasomes can be activated by toxic ALS proteins, such as TDP-43 [70]. Here, we observed inflammasome formation by the presence of a perinuclear ASC speck structure that colocalised with NLRP3 protein in a small subset of ALS MDMi (3%) (Fig. 4c). Pearson's colocalisation efficiency further confirmed this colocalisation between NLRP3 and ASC in ALS MDMi (PCC; *P* > 0.0001) compared to HC MDMi (Fig. 4d). Additionally, not all ASC foci colocalised with NLRP3, suggesting that other inflammasomes may be involved in this process. Moreover, inflammasome formation could induce pyroptosis in microglia [71]. Therefore, low inflammasome formation in ALS MDMi may reflect the ability to capture inflammasome formation before pyroptosis takes place. No colocalisation of ASC protein and NLRP3 inflammasome was observed in HC MDMi. The formation of NLRP3 inflammasome was also cell-specific and was independent of sex and the disease subgroup, indicating a broader dysregulation within ALS MDMi. Together, these results demonstrate the ability of MDMi to reflect a range of potential pathogenic pathways, which may be the key to elucidating the role of TDP-43 in ALS.

### Altered cytokine and chemokine profiles in ALS MDMi

**Fig. 5:**
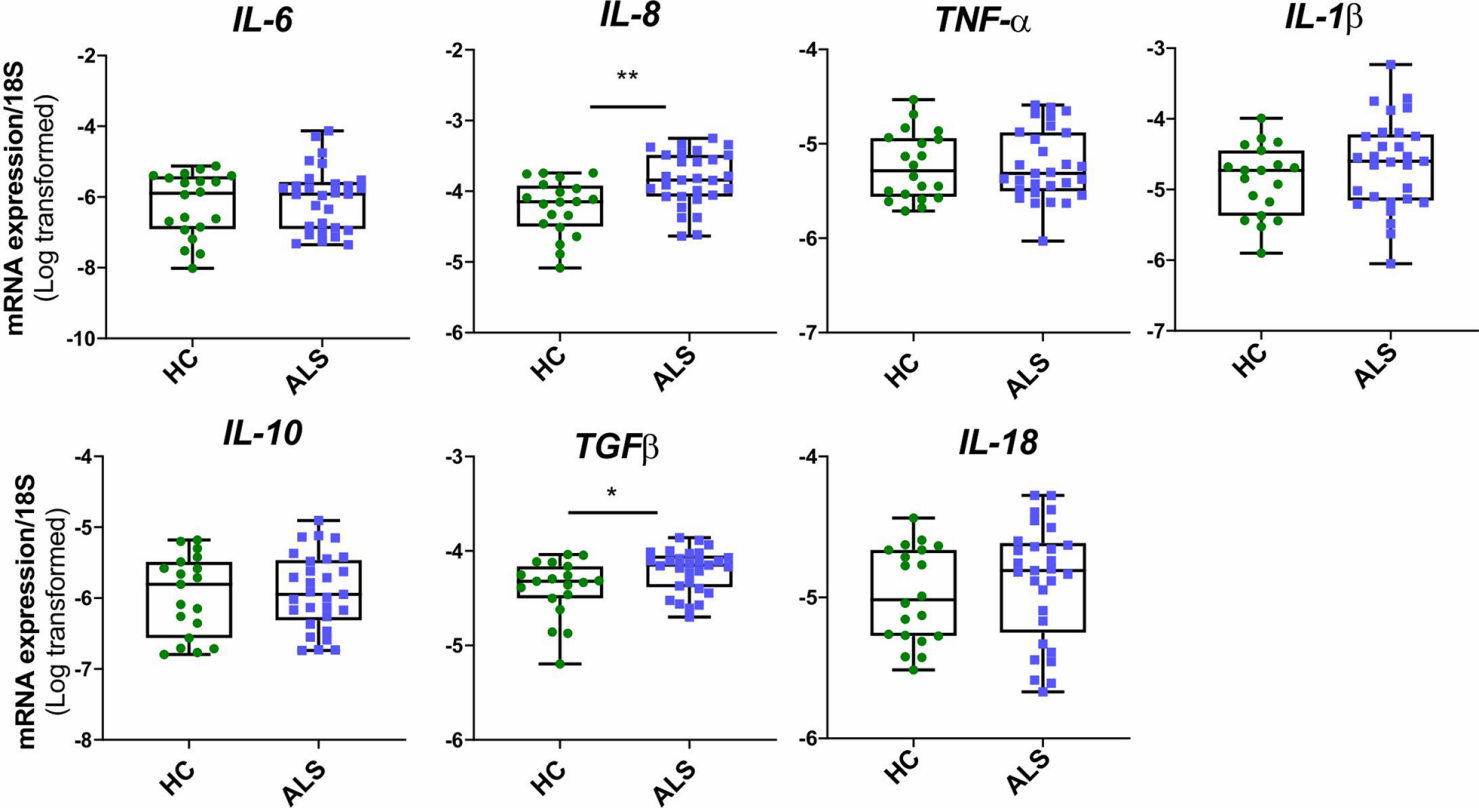
Altered cytokine mRNA expression in ALS compared to HC MDMi Box-and-whisker plot of mRNA expression of pro-inflammatory cytokines (*IL-6, IL-8, TNFα, IL-1β, IL-18*) and alternatively activated cytokines (*IL-10, TGFβ*) in HC and ALS MDMi. HC and ALS MDMi were differentiated for 14 days prior to the collection of supernatant. Data were first tested for normality using Shapiro–Wilk test. Statistical analysis between two groups was performed using Mann Whitney *U* tests. Values are the mean ± SD (*P < 0.05, **P < 0.01)

mRNA expression of cytokine profiles was determined using qPCR. Overall, an upregulation of *IL-8* expression in ALS was observed compared to HC MDMi (*P* = 0.0022) (Fig. 5). This significant upregulation was observed across each ALS subgroup (slow, *P* = 0.04, intermediate, *P* = 0.0131, rapid, *P* = 0.0034) (Additional file 1: Fig. S4). Additionally, upregulation of *TGFβ* expression in ALS MDMi was observed compared to HC. This significant upregulation was found within intermediate and rapid subgroups but not in the slow disease subgroup (Additional file 1: Fig. S4). No apparent changes were observed with other cytokines.

We next examined if the changes in mRNA expression levels were followed by similar changes in protein secretion. This was determined using a multiplex (13-plex) cytokine and chemokine immunoassay. MCP-1 (CCL2), IL-8 and IFN*α*2 were the only cytokines detectable in all HC and ALS MDMi supernatants (n = 9) (Table 2, Additional file 1: Fig. S5). A significant increase in IL-8 secretion was observed in ALS compared to HC MDMi *(P* = *0.0244)*, consistent with *IL-8* mRNA expression. An increasing trend of IFNα2, IFNγ and IL-1 family cytokines (IL-33, IL-1β and IL-18) and an unexpected decrease in MCP-1 secretion was observed in ALS MDMi compared to HC (Additional file 1: Fig. S5). This indicates that the increased levels of MCP-1, typically observed in the sera and CSF of ALS patients [76–78] could be contributed by other peripheral immune cells and/or neuroglia rather than microglia. Overall, these results indicate an altered cytokine and chemokine expression in ALS MDMi, suggesting an intrinsic role in disease progression.

**Table 2:** Cytokine and chemokines secretion levels in HC and ALS MDMi

Analyte	HC^a^	ALS^a^	*P* value^b^
IL-8 (CXCL8)	3019 (*n* = 9, ± 2402)	9763 (*n* = 9, ± 6357)	0.0244*
IFNα2	1.34 (*n* = 9, ± 0.8)	3.23 (*n* = 9, ± 2.9)	0.1903
MCP-1 (CCL-2)	35,764 (*n* = 9, ± 26,947)	5795 (*n* = 9, ± 6031)	0.077
IL-6	80.31 (*n* = 7, ± 97)	1114 (*n* = 9, ± 2152)	0.2105
IL-1β	4.37 (*n* = 2, ± 3.2)	9.58 (*n* = 8, ± 7.5)	0.2667
IL-18	2.8 (*n* = 6, ± 0.7)	18.6 (*n* = 8, ± 13.6)	0.043*
IL-33	29.0 (*n* = 3, ± 2.2)	68.4 (*n* = 9, ± 49)	0.1364
IFNγ	0.5 (*n* = 1, ± 0)	2.97 (*n* = 9, ± 3.2)	0.4913
IL-10	3.54 (*n* = 1, ± 0)	10.44 (*n* = 2, ± 6)	0.5213
IL-23	14.77 (*n* = 6, ± 5.6)	185.1 (*n* = 2, ± 96)	0.0714
TNF-α	1.8 (*n* = 6, ± 1.4)	5.76 (*n* = 3, ± 0.94)	0.0031**
IL-17A	Below limit of detection		
IL-12p70			

^a^Mean pg/ml (*n*, ± SD)

^b^Mann–Whitney *U* test, *P < 0.05, **P < 0.01

### Impaired phagocytosis in ALS MDMi

We next examined the phagocytic capability of ALS MDMi compared to HC using pHrodo-labelled *E.coli* particles (from 11 ALS and 10 HC MDMi). PHrodo-labelled *E.coli* beads was added into MDMi cultures and imaged every hour using the IncuCyte ZOOM live imaging platform.

**Fig. 6:**
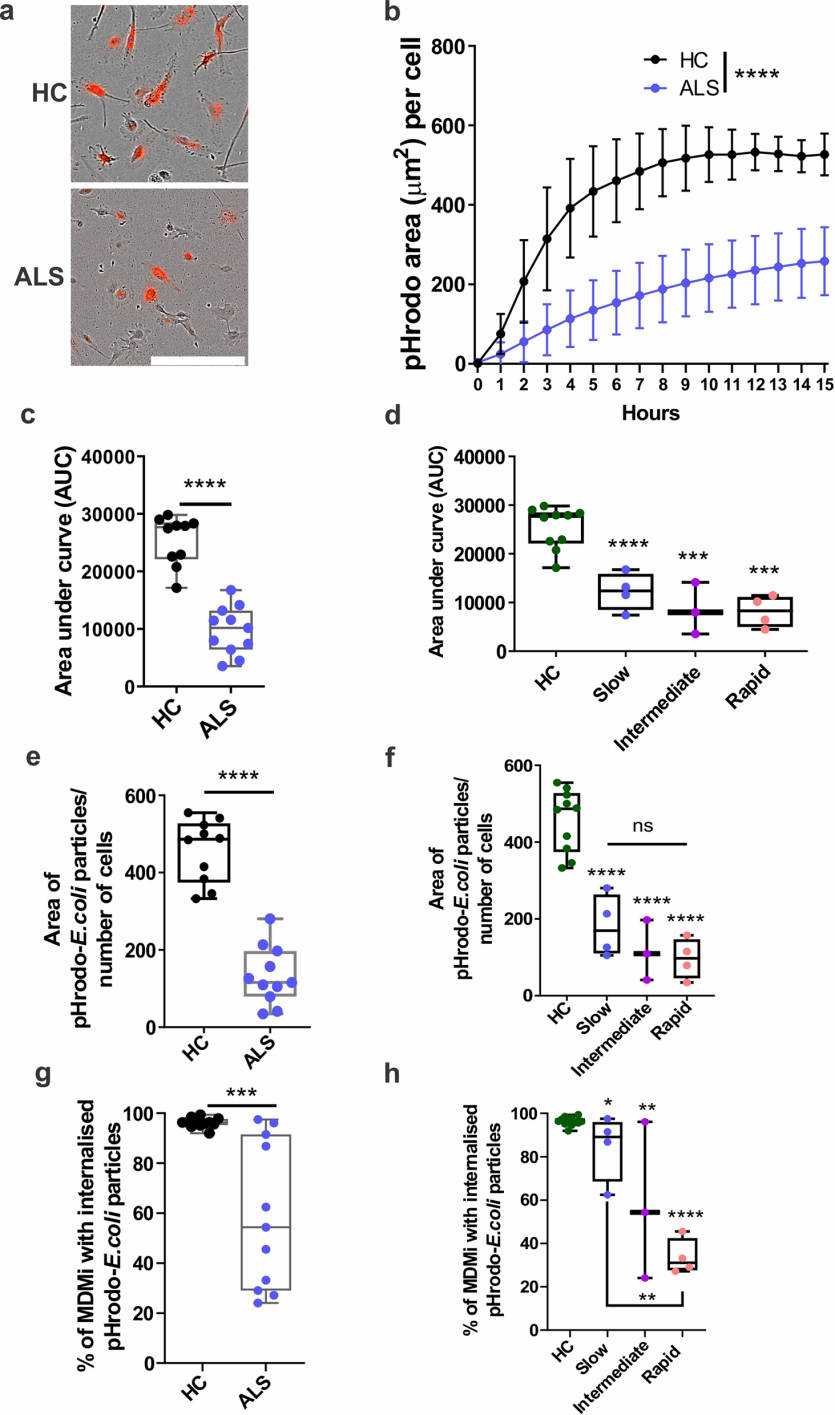
Impaired phagocytosis in ALS compared to HC MDMi.** a** Representative images of HC and ALS MDMi uptake of pHrodo-labelled *E.coli* particles (red). **b** Quantification of phagocytosis by pHrodo-labelled *E.coli* particles over 15 h using live imaging in HC and ALS. **c** Uptake of pHrodo-labelled *E.coli* particles in HC and ALS MDMi was quantified using area under the curve. **d** From (**c**), ALS MDMi were then categorised into disease subgroups. **e** Area of pHrodo-labelled *E.coli* particles normalised over cell number, quantified using IncuCyte ZOOM in-built software. **f** From (**e**), ALS MDMi were then categorised into disease subgroups. Area of pHrodo-labelled *E.coli* particles normalised against cell number, quantified using IncuCyte ZOOM in-built software. **g** Percentage of cells that contains phagocytose particles in HC and ALS MDMi. HC: *n* = 10, ALS: *n* = 11. *n* = 200 cells per patient or individual. **h** From (**g**), ALS MDMi were then categorised into disease subgroups. Percentage of cells that contain phagocytose particles in HC and ALS MDMi disease subgroups. HC: *n* = 10, ALS: *n* = 11. *n* = 200 cells per patient or individual. Formula as follows: (number of cells with particles/total number of cells)*100. Data were first tested for normality using Shapiro–Wilk test. Statistical analysis between two groups was performed using Student’s *t* test and between multiple groups using one-way ANOVA. For multiple testing, disease subgroups were compared between each other and to HC MDMi. All HC and ALS MDMi were differentiated for 14 days prior to downstream experiments. Values are mean ± SD (*P < 0.05, **P < 0.01, ***P < 0.001, ****P < 0.0001). Scale bars = 50 µm

We observed significantly decreased (2.6-fold reduction) phagocytic uptake of labelled *E.coli* particles in ALS (Fig. 6a, b) and a decreased area under the curve of pHrodobeads uptake per cell (Fig. 6c, d and Additional file 1: Fig. S6a–c). Moreover, this impairment in phagocytosis followed a trend associated with increasing disease severity with a 60% reduction in phagocytic activity in the slow disease subgroup (*P* = 0.015), 74% reduction in the intermediate disease subgroup (*P* = 0.0012), and 79% reduction in the rapid disease subgroup (*P* > 0.0001) compared to HC MDMi (Fig. 6d and Additional file 1: Fig. S6a–c).

This was further confirmed by decreased area (2.5-fold reduction) of labelled *E.coli* particle uptake per cell in (Fig. 6e), where all ALS disease subgroups showed impaired phagocytosis compared to HC MDMi (Fig. 6f). This impairment in phagocytic function appeared to be cell-specific, with some individual MDMi cells showing a substantial impairment while other cells in the same culture appeared relatively normal (Additional file 1: Fig. S6d). We examined this further and observed that 59% of ALS MDMi had internalised labelled-*E.coli* particles compared to 96% of HC MDMi (*P* = 0.0003) (Fig. 6g). Within the ALS disease subgroups, phagocytosis had a more significant impairment, where MDMi from slow (12%, *P* = 0.015), intermediate (40%, *P* = 0.0012), and rapid (70%, *P* > 0.0001) when compared to HC (Fig. 6h). Taken together, our findings indicate that a sub-population of ALS MDMi have a defective phagocytic capability, which correlates with the rate of disease progression.

## Discussion

Numerous in vitro models of ALS have been utilised to investigate the role of microglia in the pathogenesis of this disease. However, most of these studies were carried out on genetically modified animal models of ALS with limited relevance to TDP-43 neuropathology, a pathological hallmark typically observed within the post-mortem brains of ALS patients, reviewed extensively in [82]. There have been no reports of the TDP-43 neuropathology in human cellular models of microglia. In this study, we generated patient-specific MDMi from living individuals to elucidate the involvement of microglia in the pathogenesis and progression of ALS. For the first time, our results show the presence of abnormal accumulation of TDP-43 within the cytoplasm of ALS MDMi. These results confirm that TDP-43 depletion from the nucleus into the cytoplasm results in dysfunctional MDMi, which contribute to the pathogenesis of ALS. Previous reports on post-mortem brain sections from ALS patients have shown that abnormal TDP-43 persists in several pathologies [3, 4, 66, 79]. These include nucleus-to-cytoplasmic redistribution of TDP-43, phosphorylated TDP-43 and cytoplasmic inclusions of TDP-43 that appear as skein-like structures, occurring in 97% of ALS patients [4, 80]. Importantly, cytoplasmic inclusions that were positive for pTDP-43 have been found within the microglial cells of post-mortem brain sections, suggesting that other cell types apart from neurons can drive ALS pathology [81]. In keeping with clinical observations, ALS MDMi showed abnormal cytoplasmic TDP-43 localisation and the presence of various cytoplasmic inclusion bodies. We also confirmed the abnormal phosphorylation of S409/410 of TDP-43 within these TDP-43 positive inclusions in ALS MDMi, a process which could be linked to the increased cytoplasmic mislocalisation of this protein within these cells. Interestingly, the absence of pTDP-43 immunostaining was observed in some cytoplasmic inclusions, suggesting the presence of multiple phosphorylation sites [67] or the involvement of other post-translational modifications such as ubiquitination [5, 79] within the TDP-43 protein.

While clinical observations on a subgroup of ALS patients have shown cytoplasmic TDP-43 localisation in non-neuronal cell types, including fibroblasts [82], lymphomonocytes, and monocytes/macrophages [83, 84], abnormal TDP-43/pTDP-43 cytosolic inclusions (skein-like, granular) to date, have not been reported. Our data collectively suggest that the MDMi model system can reveal disease-specific features that could elucidate the patho-mechanisms of TDP-43 cytoplasmic accumulation and resulting neurodegeneration. Future studies should focus on delineating patient-specific TDP-43 pathologies in macrophages and MDMi to understand better the role of these cells in the pathogenesis of ALS.

We have observed a substantial reduction in the phagocytic capacity in a subpopulation of ALS MDMi. While this heterogeneous dysfunction was seen across all ALS subgroups, it was greatly exacerbated in the rapid subgroup, suggesting an association between phagocytic impairment in microglia and the rate of disease progression. However, the molecular basis of reduced phagocytic capacity in a subpopulation of MDMi within the same culture remains unclear but could be attributed to the population shift in monocyte subsets and their activation profiles during disease progression [85, 86]. Monocytes are predominantly divided into three subgroups, classical: CD14^++^/CD16^−^, intermediate: CD14^+^/CD16^+^, and non-classical: CD14^+^/CD16^++^ [87]. Changes in the proportions of these monocyte subsets, for example, an increased ratio of classical to non-classical monocytes, have been observed in ALS patients [85, 88].

Furthermore, CD14^++^ monocytes from ALS patients exhibited a lack of phagocytosis and altered motility [85]. Additionally, the alteration in monocyte activation profiles has been found to correlate with disease severity [88–90]. Overall, these findings suggest that ALS monocyte subsets and activation profiles are altered depending on the stage of the disease. Future studies should examine whether the different monocyte subsets are reflected in MDMi by enriching each monocyte subset prior to differentiation and/or performing single-cell RNA-sequencing analysis on ALS MDMi cultures. Importantly, these data demonstrate that the MDMi model can retain myeloid cell-specific epigenetic profiles in a patient-specific manner, recapitulating disease alterations.

Interestingly, altered ALS MDMi morphology (branch length and end-points), and increased aberrant cytoplasmic TDP-43 were exacerbated in the slow subgroup compared to intermediate and rapid subgroups. The relationship between ALS MDMi morphology, abnormal cytoplasmic TDP-43, and altered phagocytosis is currently unclear hence further studies with a larger cohort size is required.

Additionally, our ALS MDMi model revealed DNA damage and the activation of NLRP3 inflammasomes as two potential pathogenic pathways involved in disease pathogenesis. The increase in γH2AX foci and pan-nuclear γH2AX phosphorylation in ALS is likely mediated by oxidative and replication stress, in keeping with recent reports involving various ALS cell models [68, 82, 91]. Although it has been shown that TDP-43 is recruited less efficiently to DNA damage foci leading to a dysfunctional DNA damage response (DDR) [91], further studies are necessary to determine the extent of dysfunctional DDR in ALS MDMi. There is also a possibility that unresolved DDR can lead to the accumulation of cytosolic single-stranded (ss) DNA and dsDNA, in microglia and neurons, leading to neuroinflammation and subsequent cell death [48, 92, 93].

Activation of the innate immune response triggered by NLRP3 is the most common inflammasome pathway involved in neurodegeneration [94]. However, the role of NLRP3 inflammasomes in ALS remains elusive. ALS protein inclusions such as TDP-43 and SOD-1 can trigger microglial NLRP3 inflammasome activation [71]. Indeed, upregulation and activation of NLRP3 inflammasome components have been observed in ALS patients and in mouse models of ALS [73]. Inflammasome activation is a unique 2-step process that requires; 1) a priming signal for the upregulation of NLRP3 and pro-IL-1β and pro-IL-18, and 2) an activation step involving the recruitment of inflammasome adapter-ASC, activation of caspase-1 protease and the cleavage and release of mature IL-1β and IL-18 [75]. This event can lead to an inflammatory form of cell death termed pyroptosis. The co-localisation of NLRP3 protein and ASC specks observed in unstimulated ALS MDMi, suggests that the MDMi were primed. However, although increased trends of IL-1β and IL-18 cytokines were observed (Table 2 and Additional file 1: Fig. S5) in ALS MDMi, other inflammasome components such as capase-1 activity should be confirmed for complete inflammasome activation. It is unclear if priming is intracellular (cytosolic TDP-43 inclusions) and/or extracellular (alteration in cytokine and chemokine levels). Nonetheless, elevated levels of caspase 1, IL-1β and IL-18 cytokine have been reported in serum and spinal cord tissues from ALS patients, supporting the involvement of inflammasomes in ALS pathology [73].

Transcriptomic analysis of monocyte-derived microglia established in this study have been previously shown to resemble primary fetal brain microglia and iPSC-derived microglia, displaying bonafide microglial genes (*P2RY12, C1QA, MERTK, PROS1, GPR34* and *GAS6*) compared to monocyte-derived macrophages [34, 37, 42], which do not recapitulate transcriptional profiles observed in isolated adult ex vivo microglia (24 h post serum starvation), cultured brain ex vivo primary microglia [58], and in an immortalised microglial cell line [37]. This is expected as robust changes to microglia transcriptional signatures have been observed in ex vivo or cultured microglia in other published model systems [58, 95]. Numerous studies have shown that the alterations to microglial genetic signatures and functional characteristics are largely dependent on environmental conditions [58]. In keeping with this, we recently demonstrated that co-culture with the use of other CNS cell types enhance the MDMi culture model system to better recapitulate microglia within the human brain [96].

## Conclusions

**Fig. 7:**
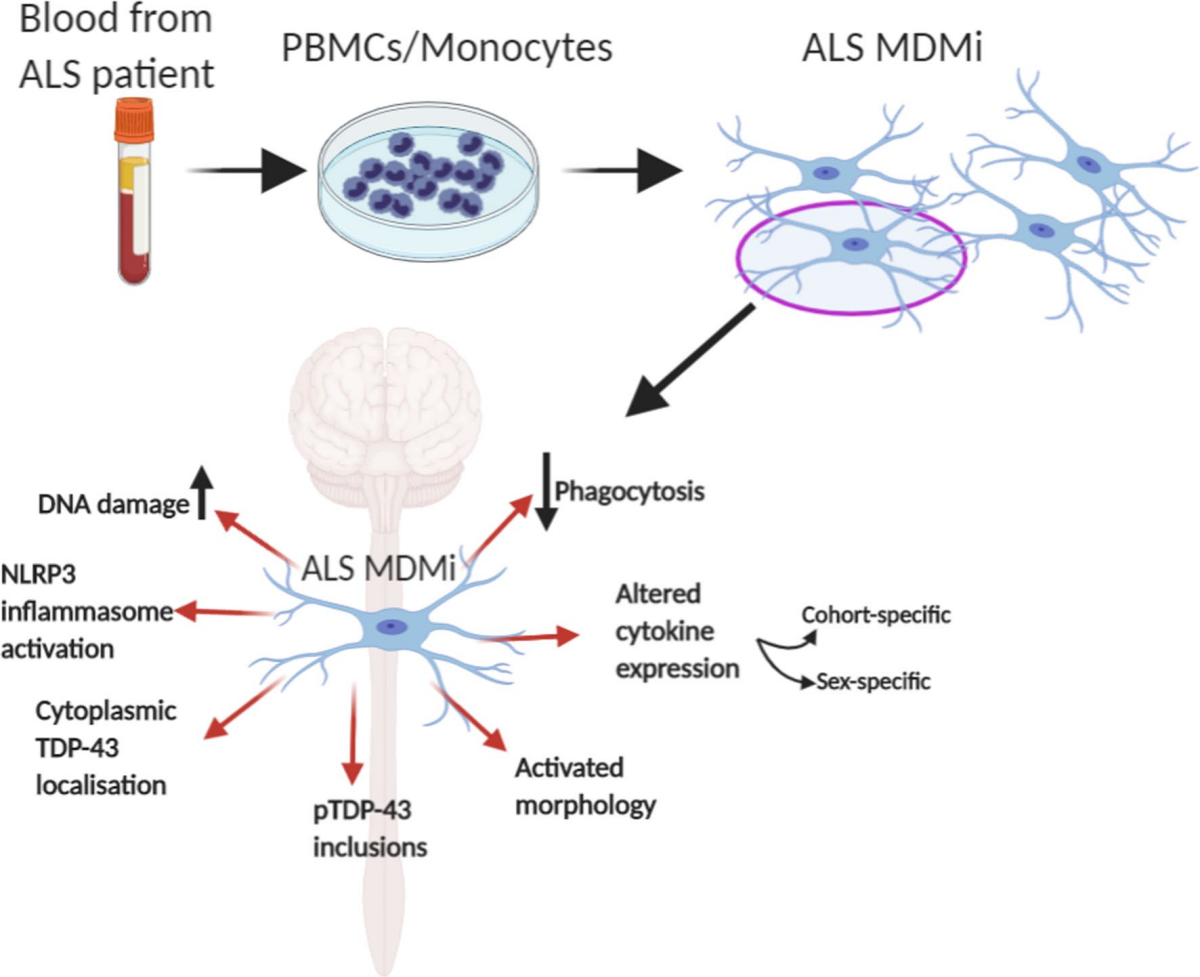
Graphic summary: Characterisation of ALS MDMi and pathological pathways for potential microglia targeted therapy. Blood monocytes derived from ALS patient PBMCs were successfully cultured to microglia-like cells (monocyte-derived microglia, MDMi). ALS MDMi recapitulated hallmarks of ALS pathology, including cytoplasmic TDP-43 localisation and phosphorylated (p)-TDP-43 inclusions. A range of abnormalities including microglial activation, altered cytokine expression, and decreased phagocytosis was observed in ALS MDMi compared to HC MDMi. MDMi model is highly suited to investigate patient heterogeneity, drug screening, and providing a basis for automated drug screening platforms in ALS and other neurodegenerative diseases

In summary, we have demonstrated that MDMi recapitulate important characteristics of microglia, such as expression of genes enriched in brain microglia, ramified morphology and phagocytic capability. Additionally, we show for the first time that ALS MDMi recapitulate key neuropathological features, including TDP-43 proteinopathies, activated microglial morphology, DNA damage, cytokine alterations, and impaired phagocytosis (Fig. 7). This unique platform is a simple and readily accessible system that could provide the basis for novel biomarkers and targeted therapeutics for the future treatment of ALS.

## Supplementary Information

**Additional file 1.** Additional tables and figures.

**Additional file 2.** Video depicts the uptake of pHrodo-labelled* E.coli* particles by MDMi.

## Data Availability

All data generated or analysed during this study are included in this published article [and its Additional file 1 and Additional file 2].

## Abbreviations

ALS: Amyotrophic lateral sclerosis
ALSFRS-R: Revised Amyotrophic Lateral Sclerosis Functional Rating Scale
CNS: Central nervous system
GM-CSF: Granulocyte–macrophage colony-stimulating factor
HC: Healthy controls
IL-34: Interleukin-34
iPSCs: Induced pluripotent stem cell
LPS: Lipopolysaccharide
MDMi: Monocyte-derived microglia-like cells (induced)
MDMa: Monocyte-derived macrophages
MDDCs: Monocyte-derived dendritic cells
NLRP3: NOD-leucine rich repeat and pyrin containing protein 3
γH2AX: Phosphorylated histone H2AX
TDP-43: TAR DNA binding protein of 43 kDA
PBMCs: Peripheral blood mononuclear cells
