# Supplementary material for: ALS monocyte-derived microglia-like cells reveal cytoplasmic TDP-43 accumulation, DNA damage, and cell-specific impairment of phagocytosis associated with disease progression
Source: J Neuroinflammation. 2022 Feb 28;19:58. doi: 10.1186/s12974-022-02421-1 (PMC8887023; doi:10.1186/s12974-022-02421-1)
Supplement: Supplementary file 1 — Additional file 1. Additional tables and figures. [file 12974_2022_2421_MOESM1_ESM.docx]

**Additional file 1**

**ALS monocyte-derived microglia-like cells reveal cytoplasmic TDP-43 accumulation, DNA damage, and cell-specific impairment of phagocytosis associated with disease progression**

Hazel Quek^1*^; Carla Cuní-López^1^; Romal Stewart^1^; Tiziana Colletti^2^; Antonietta Notaro^2^; Tam Hong Nguyen^1^; Yifan Sun^1^; Christine C. Guo^1^; Michelle K. Lupton^1^; Tara L. Roberts^3^; Yi Chieh Lim^4^; Lotta E. Oikari^1^; Vincenzo La Bella^2^; Anthony R. White^1*^

^1^ QIMR Berghofer Medical Research Institute, Brisbane, Queensland, Australia.

^2^ ALS Clinical Research Centre and Laboratory of Neurochemistry, Department of Experimental Biomedicine and Clinical Neurosciences, University of Palermo, Italy.

^3^ Ingham Institute for Applied Medical Research and School of Medicine, Western Sydney University, Liverpool, New South Wales, Australia.

^4^ Brain Tumour Biology, Danish Cancer Society, Copenhagen, Denmark

^*^Correspondence: Anthony White, QIMR Berghofer Medical Research Institute, Brisbane, Queensland, Australia. Phone: +61 410357010. Email: [Tony.White@qimrberghofer.edu.au](mailto:Tony.White@qimrberghofer.edu.au) and Hazel Quek, QIMR Berghofer Medical Research Institute, Brisbane, Queensland, Australia. Phone: +61 421926609. Email: Hazel.quek@qimrberghofer.edu.au

| **Primer** | **Forward** | **Reverse** | **Size** |
| --- | --- | --- | --- |
| *18S* | TTCGAGGCCCTGTAATTGGA | GCAGCAACTTTAATATACGCTATTGG | 123bp |
| *APOE* | GTTGCTGGTCACATTCCTGG | GCAGGTAATCCCAAAAGCGAC | 146bp |
| *P2RY12* | AAGAGCACTCAAGACTTTAC | GGGTTTGAATGTATCCAGTAAG | 147bp |
| *PU.1* | AGCAGATGCACGTCCTCGATA | AGACCTGGTGGCCAAGACTG | 63bp |
| *IRF8* | AGGAGCCTTCTGTGGACGAT | GGGAGAATGCTGAATGGTGC | 168bp |
| *RUNX1* | TCGCAGCGTGGTAAAAGAAA | GCACTGTGGGTACGAAGGAA | 117bp |
| *CX3CR1* | CTTACGATGGCACCCAGTGA | CAAGGCAGTCCAGGAGAGTT | 79bp |
| *CD68* | CTTCTCTCATTCCCCTATGGACA | GAAGGACACATTGTACTCCACC | 105bp |
| *CD45* | GCAGCTAGCAAGTGGTTTGTTC | AAACAGCATGCGTCCTTTCTC | 92bp |
| *C1QA* | ATGGTGACCGAGGACTTGTG | GTCCTTGATGTTTCCTGGGC | 276bp |
| *PROS1* | TTGCACTTGTAAACCAGGTTGG | CAGGAACAGTGGTAACTTCCAG | 132bp |
| *GAS6* | CTCTCTCTGTGGCACTGGTA | CCTTGATCTCCATTAGGGCCAA | 105bp |
| *MERTK* | CTCTGGCGTAGAGCTATCACT | AGGCTGGGTTGGTGAAAACA | 162bp |
| *GPR34* | CCTGATGTCCAGTAACATTCGC | CATGCAGGGAGTATCCTGGT | 116bp |
| *TREM2* | TCTTTGTCACAGAGCTGTCC | TCATAGGGGCAAGACACCTG | 88bp |
| *HLA-DRA* | CCCAGGGAAGACCACCTTT | CACCCTGCAGTCGTAAACGT | 81bp |
| *CD209* | AATGGCTGGAACGACGACAAA | CAGGAGGCTGCGGACTTTTT | 68bp |
| *CCR7* | GGGGAAACCAATGAAAAGC | ACCTCATCTTGACACAGGCATA | 75bp |
| *CD206* | TGCAGAAGCAAACCAAACCTGTAA | CAGGCCTTAAGCCAACGAAACT | 104bp |
| *Ki67* | gaggtgtgcagaaaatccaaa | ctgtccctatgacttctggttgt | 78bp |
| *IL-6* | TGCAATAACCACCCCTGACC | TGCGCAGAATGAGATGAGTTG | 104bp |
| *IL-8* | AGACAGCAGAGCACACAAGC | ATGGTTCCTTCCGGTGGT | 62bp |
| *IL-10* | TGCTGGAGGACTTTAAGGGTTA | GATGCCTTTCTCTTGGAGCTTA | 254bp |
| *TNF-α* | CAGCCTCTTCTCCTTCCTGAT | GCCAGAGGGCTGATTAGAGA | 123bp |
| *TGF-β* | CGCGTGCTAATGGTGGAAA | CTCGGAGCTCTGATGTGTTGAA | 97bp |
| *IL-1β* | AATCTGTACCTGTCCTGCGTGTT | TGGGTAATTTTTGGGATCTACACTCT | 78bp |
| *IL-18* | AACAAACTATTTGTCGCAGGAAT | TGCCACAAAGTTGATGCAAT | 72bp |

**Additional file 1: Table 1.** Primers used for qPCR analysis

*Gene names are in italic.

| **Culture conditions** | **Quek *et al*** | **Ohgidani *et al*., 2014** | **Sellgren *et al*., 2017** |
| --- | --- | --- | --- |
| **Cell source** | Blood-derived monocytes | Blood-derived monocytes | Blood-derived monocytes |
| **Medium** | RPMI GlutaMAX | RPMI GlutaMAX | RPMI GlutaMAX |
| **Supplements** | GM-CSF (10ng/ml)  IL-34 (100ng/ml) | GM-CSF (10ng/ml)  IL-34 (100ng/ml) | GM-CSF (10ng/ml)  IL-34 (100ng/ml) |
| **Days in culture** | 14 | 14 | 11 |
| **Seeding density** | 4x10^5^cells/well  48-well plate | 4x10^5^ cells/mL | 5x10^5^cells/well  24-well plate |
| **Coating** | Matrigel™ | Not reported | Geltrex™ |
| **Disease modelled** | ~~ALS~~ Amyotrophic lateral sclerosis | Nasu-Hakola disease | Schizophrenia |

**Additional file 1: Table 2.** Modifications in the MDMi differentiation protocol used in this study compared to the original protocol from Ohgidani *et al*., 2014, and a relevant protocol from Sellgren *et al*., 2017.


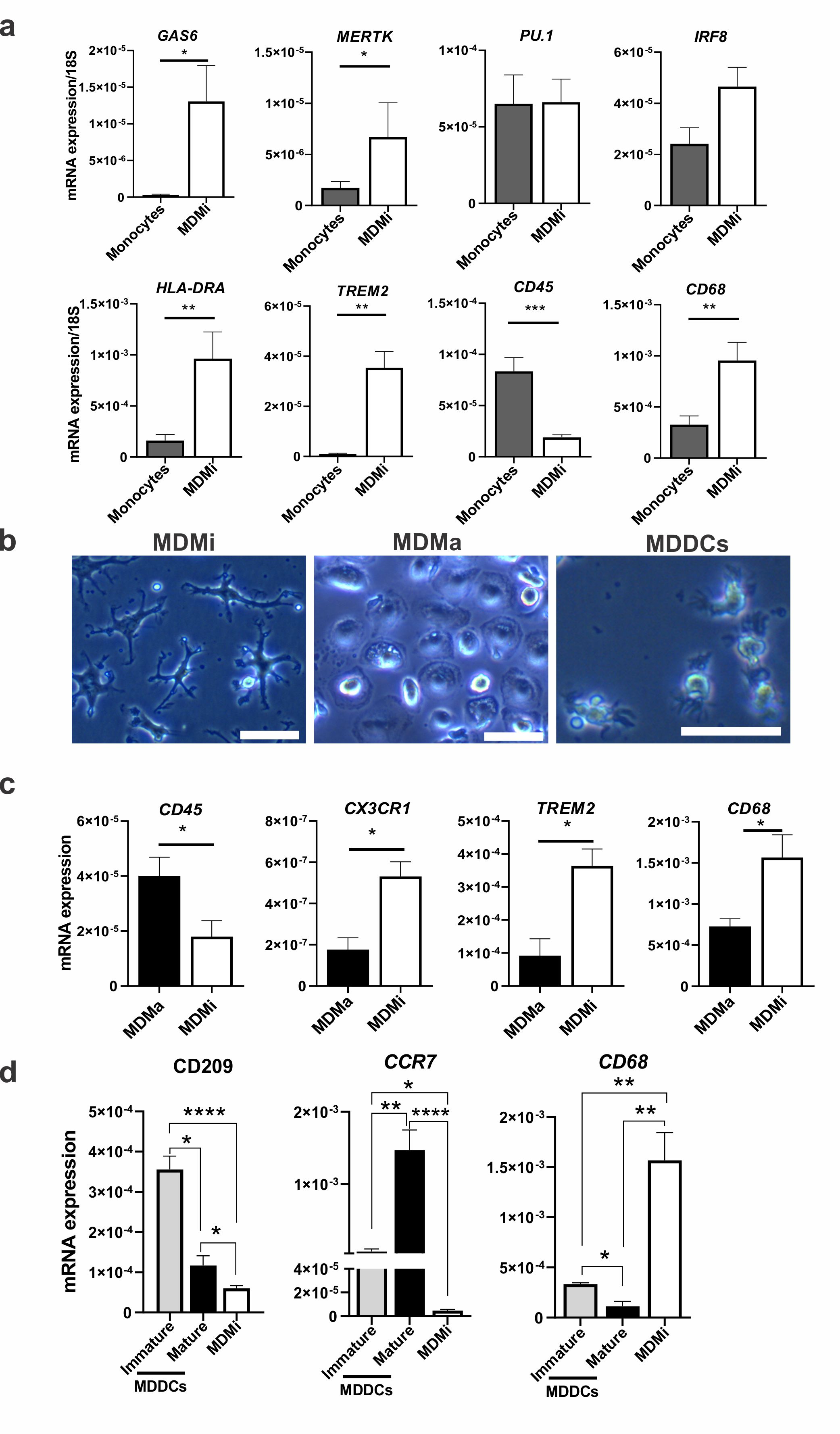


**Additional file 1: Figure 1. Gene expression analysis of MDMi with isolated monocytes, monocyte-derived macrophages (MDMa), and monocyte-derived dendritic cells (MDDCs).** MDMi, MDMa, and MDDCs were generated from matched monocytes isolated from the same healthy young volunteers (<40 years of age) to examine gene changes. **(a)** Gene expression levels of well-known published microglial genes^[5, 14-16]^ such as *GAS6, MERTK, PU.1, IRF8, HLA-DR, TREM2,* and *CD68* between isolated monocytes and day 14 MDMi. A monocyte/macrophage gene, *CD45* confirmed a monocyte phenotype. (Monocytes and MDMi from <40 years of age: n=5). **(b)** Representative phase contrast pictures of MDMi (Day 14), MDMa (Day 14), and MDDCs (Day 9). **(c)** Gene expression levels of well-characterised microglial genes *TREM2, CX3CR1* and *CD68*, and monocyte/macrophage gene, *CD45* between MDMa and MDMi at day 14. (MDMa and MDMi from <40 years of age: n=5). **(d)** Bar graph showing gene expression levels of dendritic cell markers *CD209* and *CCR7* and microglial marker, *CD68* between MDDCs and MDMi. (MDDCs and MDMi from <40 years of age: n=5). Data were first tested for normality using Shapiro-Wilk test. Statistical analysis between two groups was performed using Student’s *t* test and between multiple groups using one-way ANOVA. Values are mean ± SEM (**P* < 0.05, ** *P* < 0.01, *** *P* < 0.001, **** *P* < 0.0001). Scale bars= 50µm.


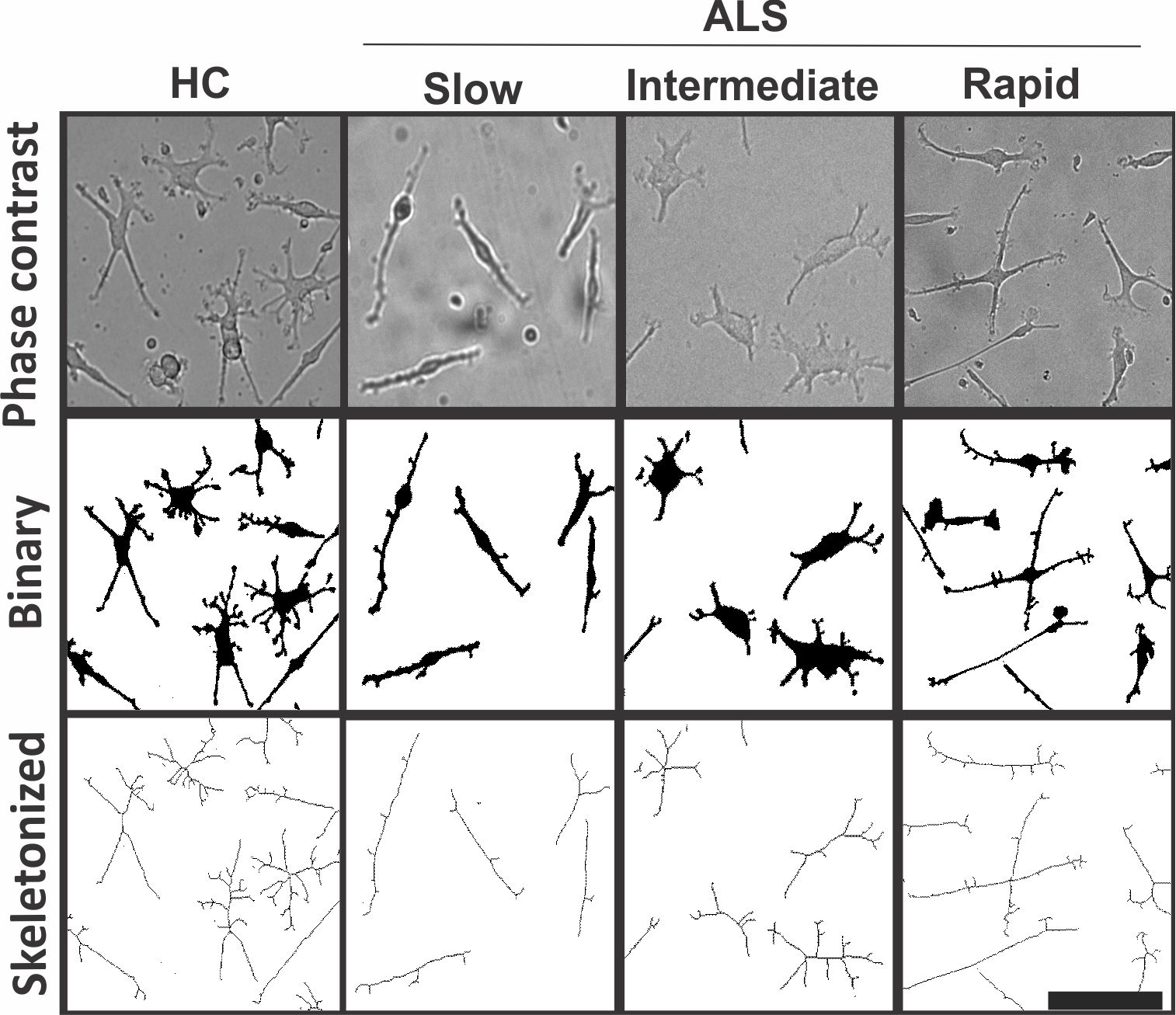


**Additional file 1: Figure 2. Morphology of ALS MDMi.** Representative images of analysed MDMi morphology in ALS subgroups at Day 14 using ImageJ. Phase contrast images were adjusted to 16-bit and thresholded to create a mask for MDMi cells. Images were then converted to binary and analysed using the AnalyzeSkeleton plugin to obtain various microglial branch information. Scale bar= 50µm.


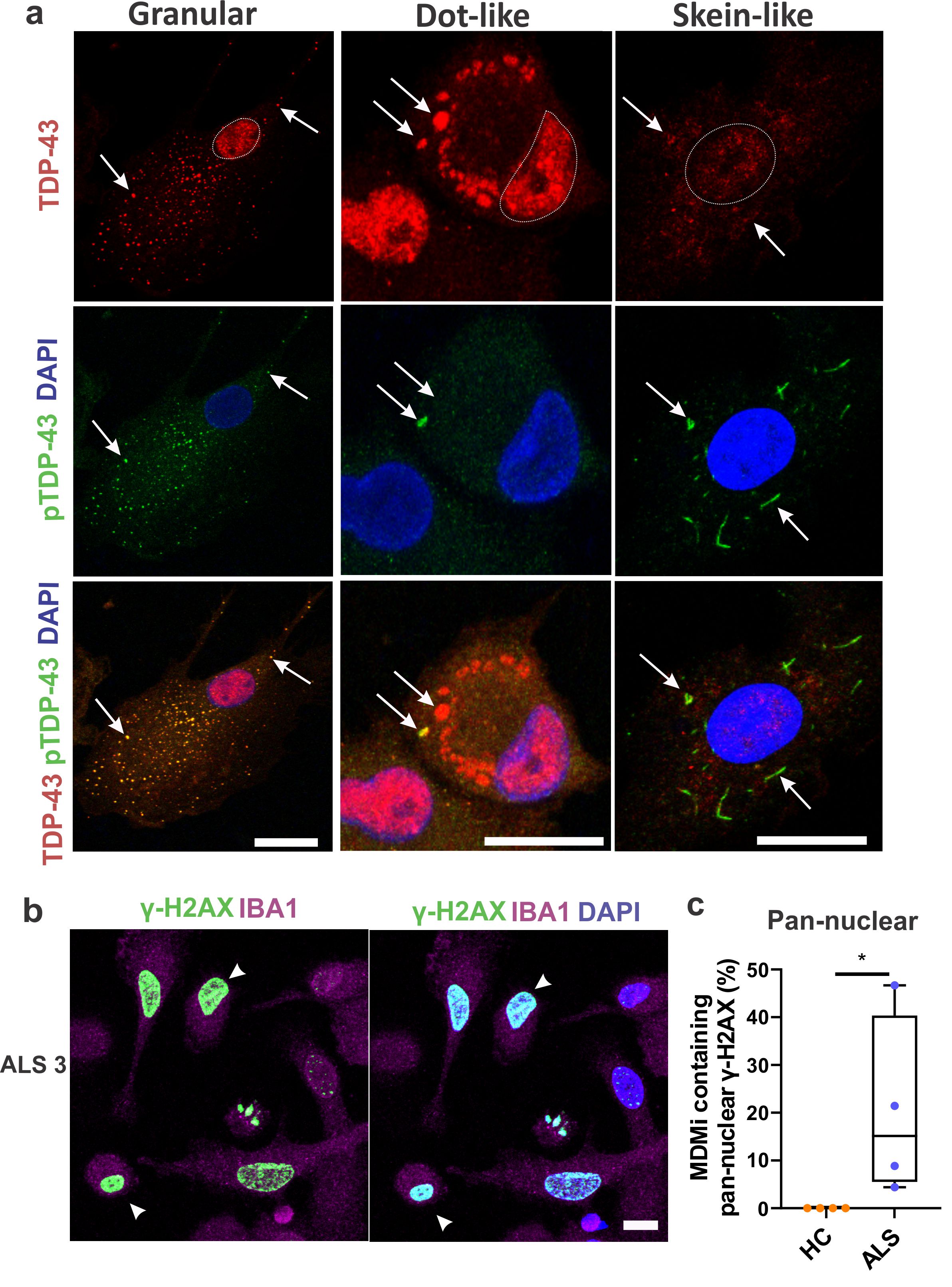


**Additional file 1: Figure 3. Day 14 ALS MDMi display abnormal TDP-43 localisation and DNA damage. (a)** Representative immunofluorescence images of HC and ALS MDMi showing TDP-43 (red), pTDP-43 (green), and DAPI counterstain (blue). White arrows indicate TDP-43 and/or pTDP-43 cytoplasmic inclusions in ALS MDMi. White arrows indicate TDP-43 positive inclusions observed in ALS MDMi including granular, dot-like, and skein-like. **(b)** Representative immunofluorescence images of ALS MDMi showing γH2AX (green), Iba1 (magenta), and DAPI counterstain (blue). Dotted white arrows indicate pan-nuclear γH2AX staining in ALS MDMi. **(c)** An increased percentage of MDMi containing pan-nuclear γH2AX phosphorylation was observed in ALS compared to HC MDMi, n=4, 60 cells per individual. Formula is as follows (number of cells containing pan-nuclear γH2AX / total number of cells)*100. Data were first tested for normality using Shapiro-Wilk test. Statistical analysis between two groups was performed using Student’s *t* test. Scale bars= 50µm. Values are mean ± SD (**P* < 0.05). Scale bars= 50µm.


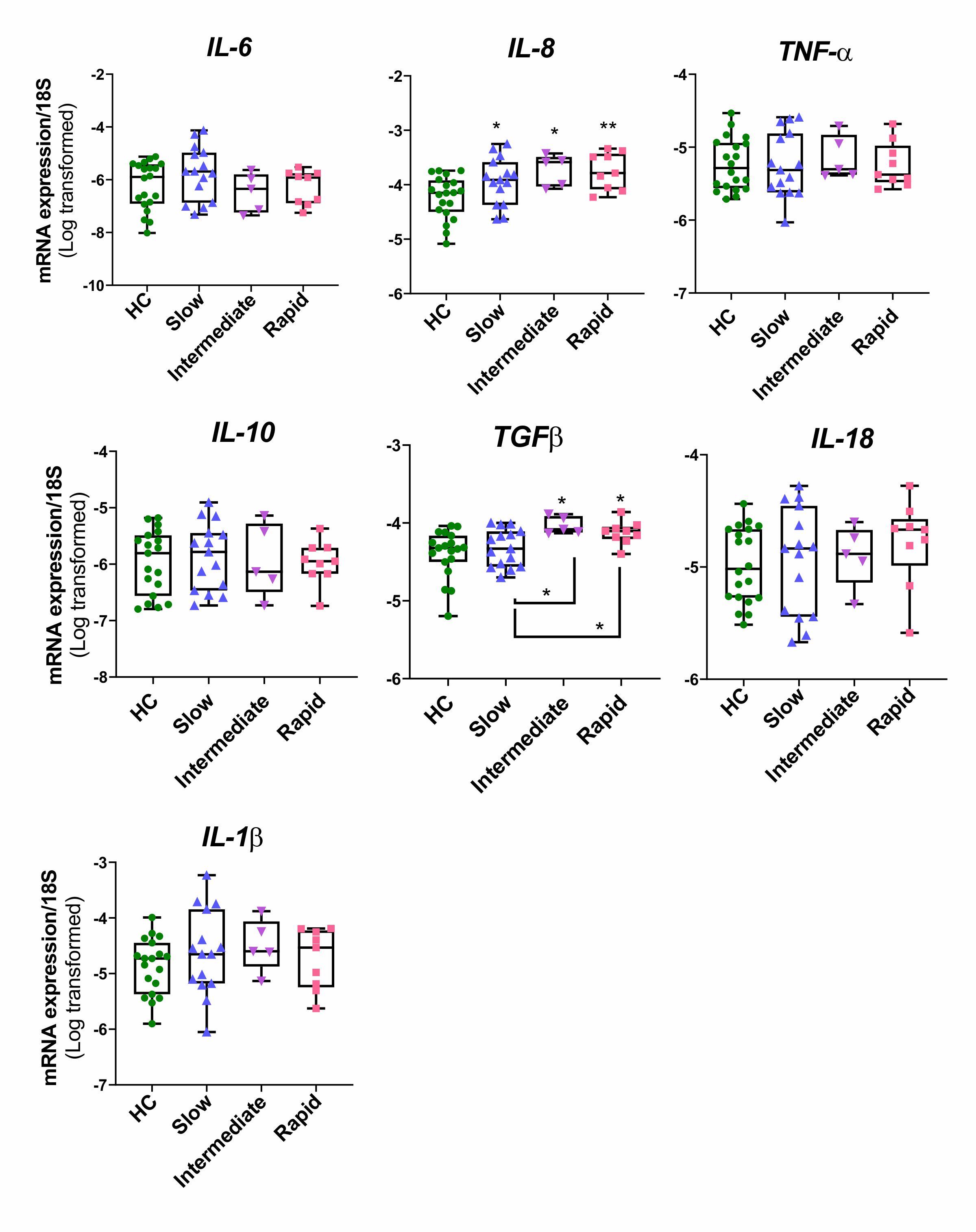


**Additional file 1: Figure 4.** ALS MDMi subgroups. HC: n=19, ALS: n=29 (Slow: n=15, Intermediate: n=5, Rapid: n=9). Supernatant was collected from HC and ALS MDMi at Day 14. Data were first tested for normality using Shapiro-Wilk test. Statistical analysis between two groups was performed using Mann Whitney *U* tests. Values are the mean ± SD (**P* < 0.05, ** *P* < 0.01).

**Additional file 1: Figure 5. Altered cytokine and chemokine secretion in ALS compared to HC MDMi.** Levels of cytokine and chemokine secretion measured using the multiplex LEGENDplex^TM^ Human Inflammation Panel 1 (13-plex) from HC and ALS MDMi supernatants collected on Day 14. All samples were below the LOD for both IL-12p70 and IL-17A and were excluded. Samples below LOD were undetectable and therefore excluded in the remaining cytokines. Only IL-8, MCP-1 and IFNα2 cytokines were detectable in all samples. HC: n=9, ALS: n=9. N for the remaining cytokines are shown in Table 2. Data were first tested for normality using Shapiro-Wilk test. Statistical analysis between two groups was performed using Mann-Whitney *U*- test. Values are mean ± SD (**P* < 0.05).


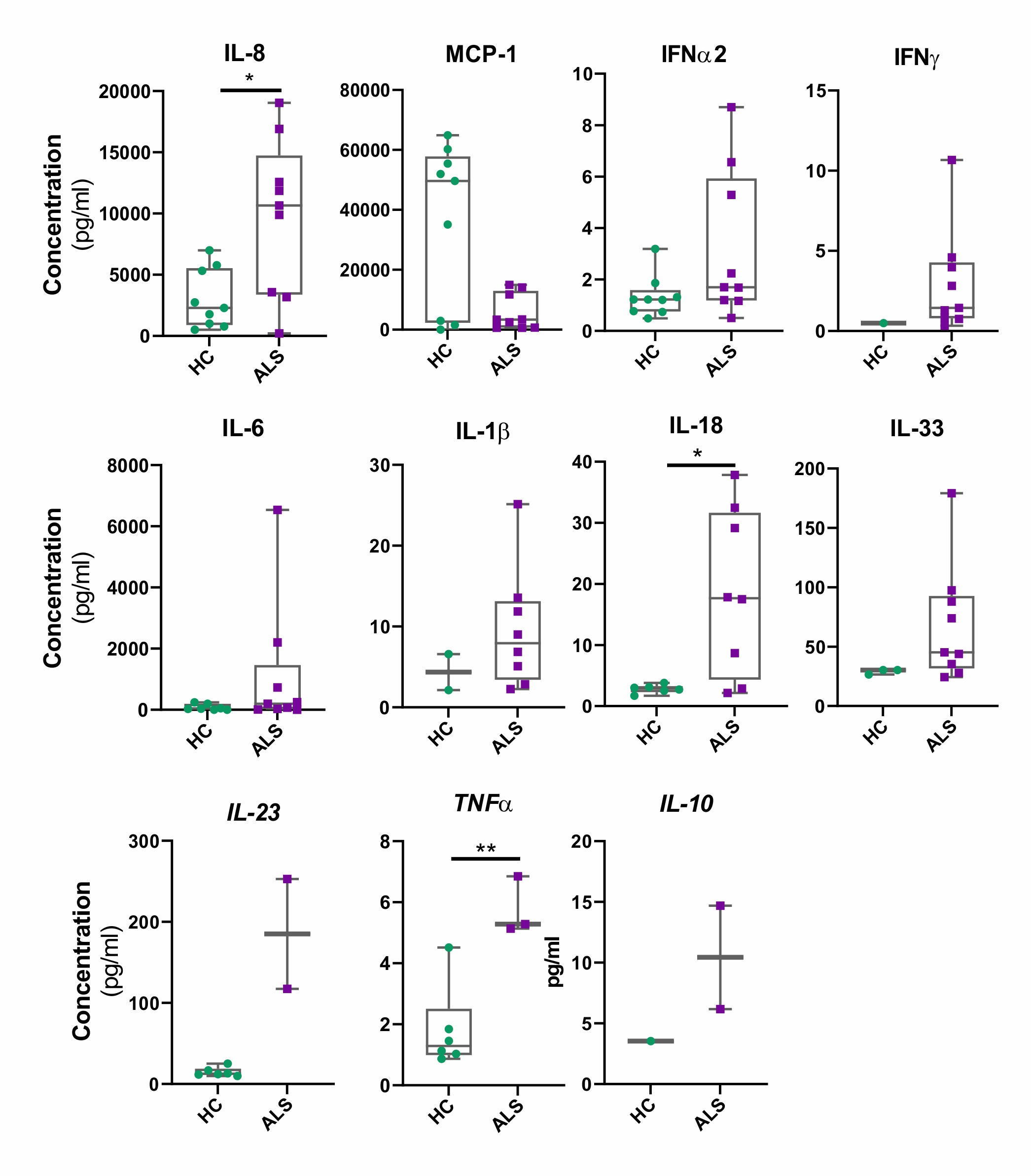

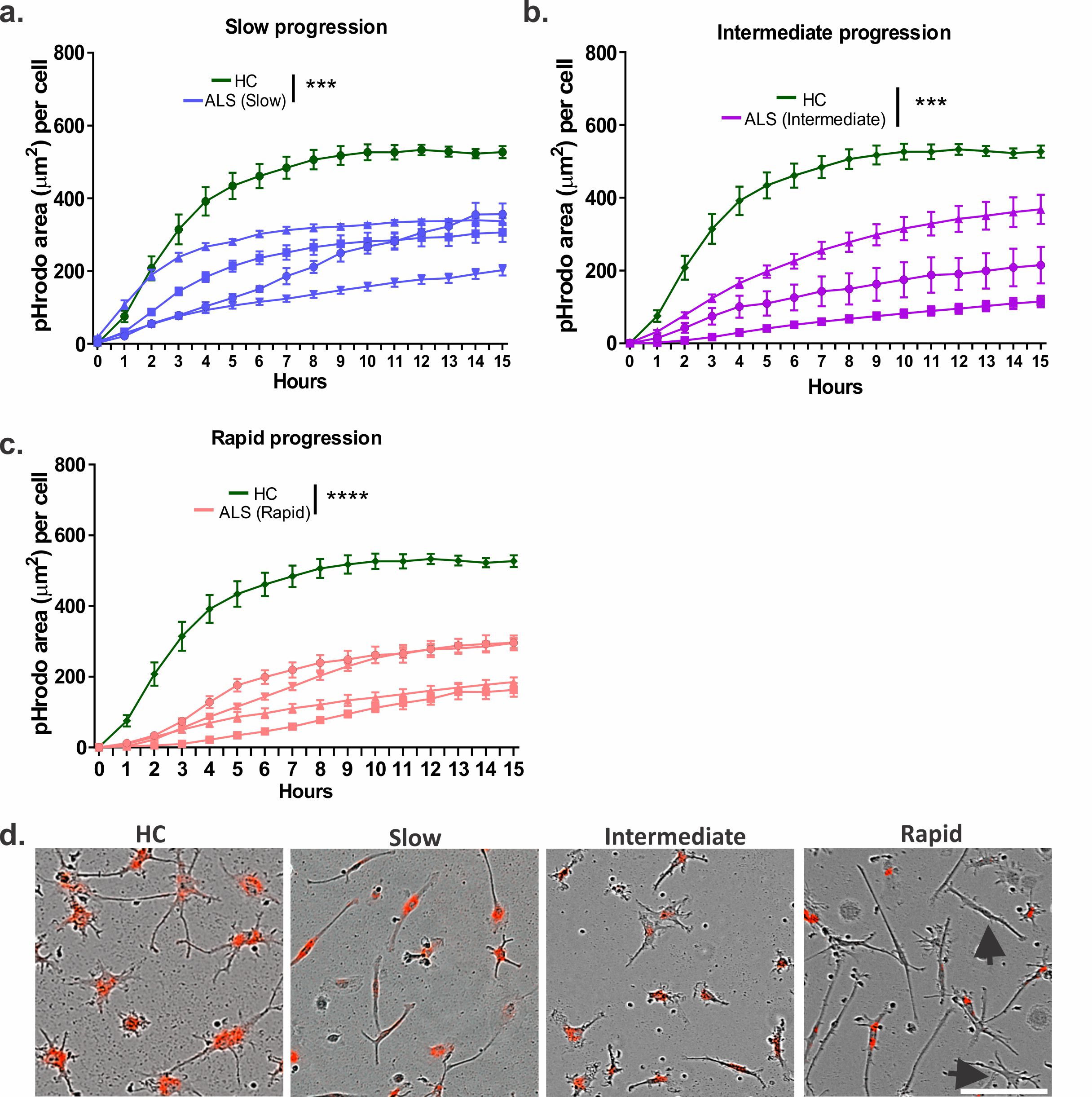


**Additional file 1: Figure 6. Impaired phagocytosis in ALS MDMi.** Quantification of pHrodo-labelled *E.coli* particles phagocytosis over 15 hrs using live imaging in HC: n=10 (green) and **(a)** ALS Slow: n=4 (blue), **(b)** Intermediate: n=3 (purple), **(c)** Rapid: n=4 (orange). **(d)** Representative images of pHrodo-labelled *E.coli* particles (red) uptake by HC and ALS subgroup MDMi. Arrows show MDMi cells that lack of pHrodo-labelled *E.coli* particles. Data were first tested for normality using Shapiro-Wilk test. Statistical analysis between two groups was performed using Student’s *t* test. Values are mean ± SEM (***p < 0.001, ****p < 0.0001). Scale bar= 50µm.
